# Shared and Distinct Neural Bases of Large- and Small-Scale Spatial Ability: A Coordinate-Based Activation Likelihood Estimation Meta-Analysis

**DOI:** 10.3389/fnins.2018.01021

**Published:** 2019-01-10

**Authors:** Yuan Li, Feng Kong, Ming Ji, Yangmei Luo, Jijun Lan, Xuqun You

**Affiliations:** ^1^School of Psychology, Shaanxi Normal University, Xi'an, China; ^2^Shaanxi Provincial Key Laboratory of Behavior and Cognitive Neuroscience, Xi'an, China

**Keywords:** large-scale spatial ability, small-scale spatial ability, activation likelihood estimation, meta-analysis, behavioral model

## Abstract

**Background:** Spatial ability is vital for human survival and development. However, the relationship between large-scale and small-scale spatial ability remains poorly understood. To address this issue from a novel perspective, we performed an activation likelihood estimation (ALE) meta-analysis of neuroimaging studies to determine the shared and distinct neural bases of these two forms of spatial ability.

**Methods:** We searched Web of Science, PubMed, PsycINFO, and Google Scholar for studies regarding “spatial ability” published within the last 20 years (January 1988 through June 2018). A final total of 103 studies (**Table 1**) involving 2,085 participants (male = 1,116) and 2,586 foci were incorporated into the meta-analysis.

**Results:** Large-scale spatial ability was associated with activation in the limbic lobe, posterior lobe, occipital lobe, parietal lobe, right anterior lobe, frontal lobe, and right sub-lobar area. Small-scale spatial ability was associated with activation in the parietal lobe, occipital lobe, frontal lobe, right posterior lobe, and left sub-lobar area. Furthermore, conjunction analysis revealed overlapping regions in the sub-gyrus, right superior frontal gyrus, right superior parietal lobule, right middle occipital gyrus, right superior occipital gyrus, left inferior occipital gyrus, and precuneus. The contrast analysis demonstrated that the parahippocampal gyrus, left lingual gyrus, culmen, right middle temporal gyrus, left declive, left superior occipital gyrus, and right lentiform nucleus were more strongly activated during large-scale spatial tasks. In contrast, the precuneus, right inferior frontal gyrus, right precentral gyrus, left inferior parietal lobule, left supramarginal gyrus, left superior parietal lobule, right inferior occipital gyrus, and left middle frontal gyrus were more strongly activated during small-scale spatial tasks. Our results further indicated that there is no absolute difference in the cognitive strategies associated with the two forms of spatial ability (egocentric/allocentric).

**Conclusion:** The results of the present study verify and expand upon the theoretical model of spatial ability proposed by Hegarty et al. Our analysis revealed a shared neural basis between large- and small-scale spatial abilities, as well as specific yet independent neural bases underlying each. Based on these findings, we proposed a more comprehensive version of the behavioral model.

## Introduction

Spatial ability is a core cognitive function that plays a significant role in individual intelligence. In general, spatial ability has been defined as the ability to understand the relationships among different positions in space or imagined movements of two- and three-dimensional objects (Clements, 1999; Wang et al., 2014). Specifically, spatial ability can be divided into two major categories: large-scale spatial ability and small-scale spatial ability. Large-scale spatial ability refers to the ability of individuals to carry out cognitive processing of spatial information in the large-scale environment. During this process, the viewer's perspective changes with respect to the larger environment, but the spatial relationships among individual objects remain the same (Hegarty and Waller, 2004; Wang et al., 2014). Representative examples of large-scale spatial ability include navigation and spatial orientation abilities (Jansen, 2009; Höffler, 2010; Wang et al., 2014). Navigation ability refers to the ability to navigate within a large-scale environment in which the spatial relationships among landmarks cannot be fully apprehended from a single vantage point (Wang and Carr, 2014), while spatial orientation refers to the ability to imagine objects from different perspectives (Yilmaz, 2009; Turgut, 2015).

Small-scale spatial ability can be defined as the ability to mentally represent and transform two- and three-dimensional images that can typically be apprehended from a single vantage point (Wang and Carr, 2014). Small-scale spatial ability mainly refers to spatial visualization and spatial relations capabilities (Jansen, 2009; Höffler, 2010). Spatial visualization is defined as the ability to manipulate complex spatial information involving the configurations of shapes (e.g., image folding or movement) or to mentally transform a two-dimensional object into three-dimensional object (Linn and Petersen, 1985; Yang and Chen, 2010). Spatial relations ability refers to the ability to recognize the relationships among the visual components of an object (Bosnyak and Nagy-Kondor, 2008; Turgut, 2015).

Currently, the relationship between large- and small-scale spatial ability can be explained using four models: (1) The “unitary model” assumes that spatial abilities along the two scales exhibit complete overlap, (2) while the “partial dissociation model” proposes that the two types of spatial ability exhibit similarities and differences. (3) In contrast, the “total dissociation model” assumes that the two sets of abilities are distinct. Lastly, (4) the “mediation model” assumes that small- and large-scale spatial abilities can be dissociated, but are determined by a third variable (Hegarty et al., 2006; Jansen, 2009). That is, while Models 1 and 2 propose that the two forms of spatial ability are related, Models 3, and 4 propose that they are not.

Each of the aforementioned models has been verified to some extent in previous studies. For instance, Hegarty and Waller (2004) observed a dissociative relationship between mental rotation and perspective-taking spatial abilities. A meta-analysis by Wang et al. (2014) further suggested that small- and large-scale spatial abilities are best characterized as separate entities. However, other studies have yielded conflicting results. Kozhevnikov et al. (2006) demonstrated that spatial navigation performance can be predicted based on mental rotation ability. In addition, Malinowski (2001) reported that mental rotation skills are significantly correlated with wayfinding performance during an orienteering task.

**Figure 1:**
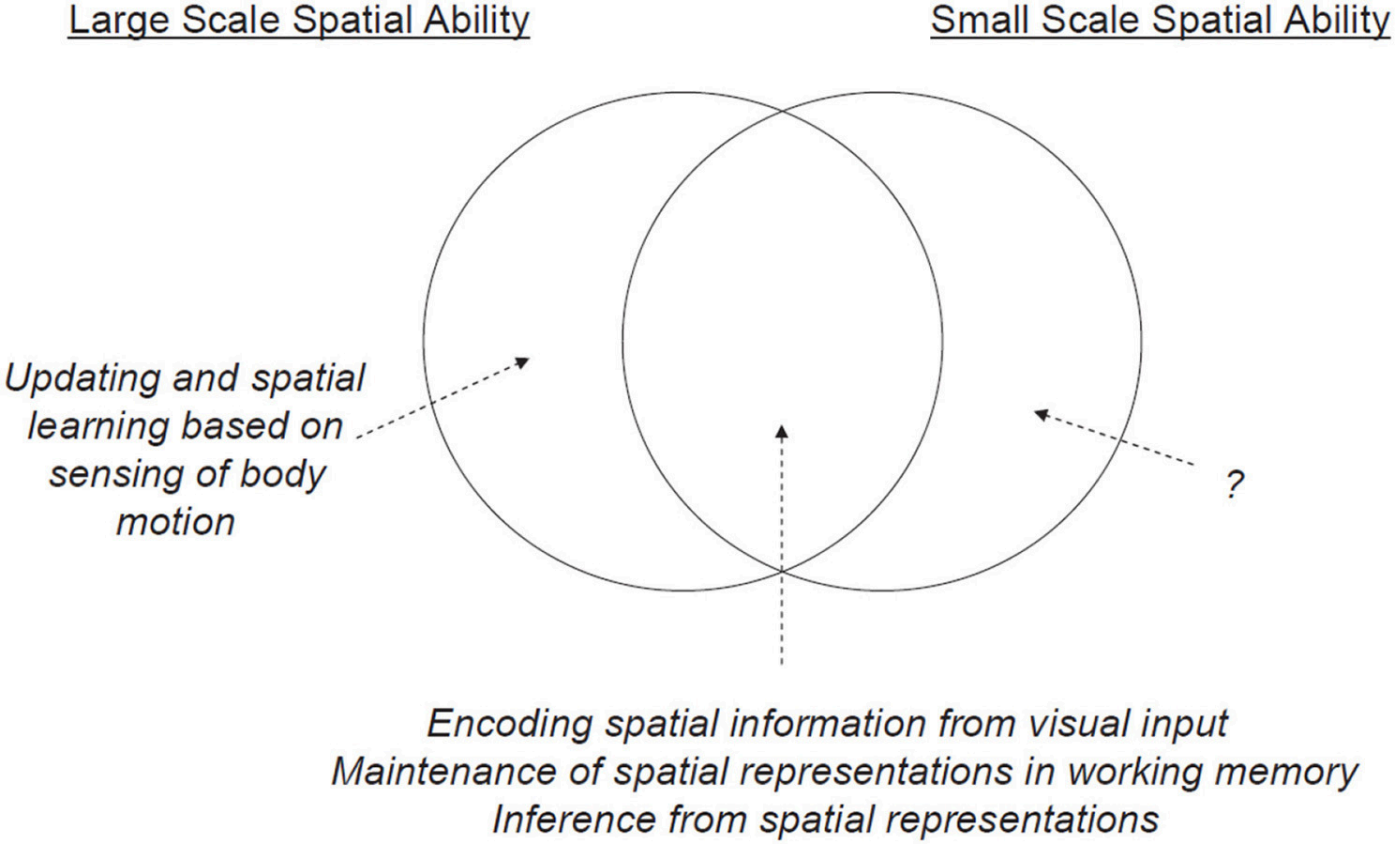
Model characterizing the relationship between large- and small-scale spatial abilities, as proposed by Hegarty et al. (2006).

Notably, in a study that examined spatial abilities, spatial updating, verbal abilities, and working memory in 221 participants, Hegarty et al. (2006) reported results that were consistent with the partial dissociation model only. Moreover, they specified the degree of overlap between small-scale and large-scale abilities, providing new insights regarding the similarities, and differences between these abilities (see Figure 1). Although their work is of great significance, the partial dissociation model remains problematic for two reasons: The model is currently incomplete, and evidence for the neural basis of such a model requires further and more comprehensive verification.

The rapid development of neuroimaging techniques in recent years has prompted extensive investigation of large- and small-scale spatial abilities using functional magnetic resonance imaging (FMRI) and positron emission tomography (PET). In an effort to verify and expand the model of spatial ability proposed by Hegarty et al. (2006), the present study aimed to elucidate the neural basis of large- and small-scale spatial abilities via activation likelihood estimation (ALE) analysis (Eickhoff et al., 2012).

## Materials and Methods

### Literature Search and Study Selection

We searched Web of Science, PubMed, PsycINFO, and Google Scholar for studies regarding “spatial ability” published within the last 20 years (January 1988 through June 2018). In order to retrieve the maximum number of relevant articles, we classified the search keywords into the following four series based on the type of spatial ability, yielding a total of 76 groups:

1. Spatial ability/Spatial cognition/Spatial perception/Spatial information processing + FMRI/PET.
2. Large-scale Spatial ability/Small-scale Spatial ability + FMRI/PET.
3. Navigation/Spatial orientation/Spatial visualization/ Spatial relations + FMRI/PET.
4. Navigation task/test + FMRI/PET; Draw maps task/test + FMRI/PET; Way-finding task/test + FMRI/PET; Map learning task/test + FMRI/PET; Spatial orientation task/test + FMRI/PET; Perspective taking task/test + FMRI/PET; Spatial visualization task/test + FMRI/PET; Mental rotations task/test + FMRI/PET; Paper folding task/test + FMRI/PET; Spatial relations task/test + FMRI/PET; Water level task/test + FMRI/PET; Card rotation task/test + FMRI/PET; Figures task/test + FMRI/PET; Differential aptitude task/test + FMRI/PET.

After four rounds of the above search process, a total of 826 documents were identified. We then examined each of the documents in full, incorporating those with the following characteristics into our meta-analysis:

1. All participants were healthy individuals.
2. The study must have included whole-brain rather than region-of-interest analyses, and the data reported must have been standardized (Montreal Neurological Institute (MNI) or Talairach space).
3. The study must have utilized behavioral experimental methods, and the articles must have included brain imaging data obtained from individuals following independent experimental tasks.
4. If an experimental result was reported in multiple papers, only one instance was utilized for the meta-analysis.

**Figure 2:**
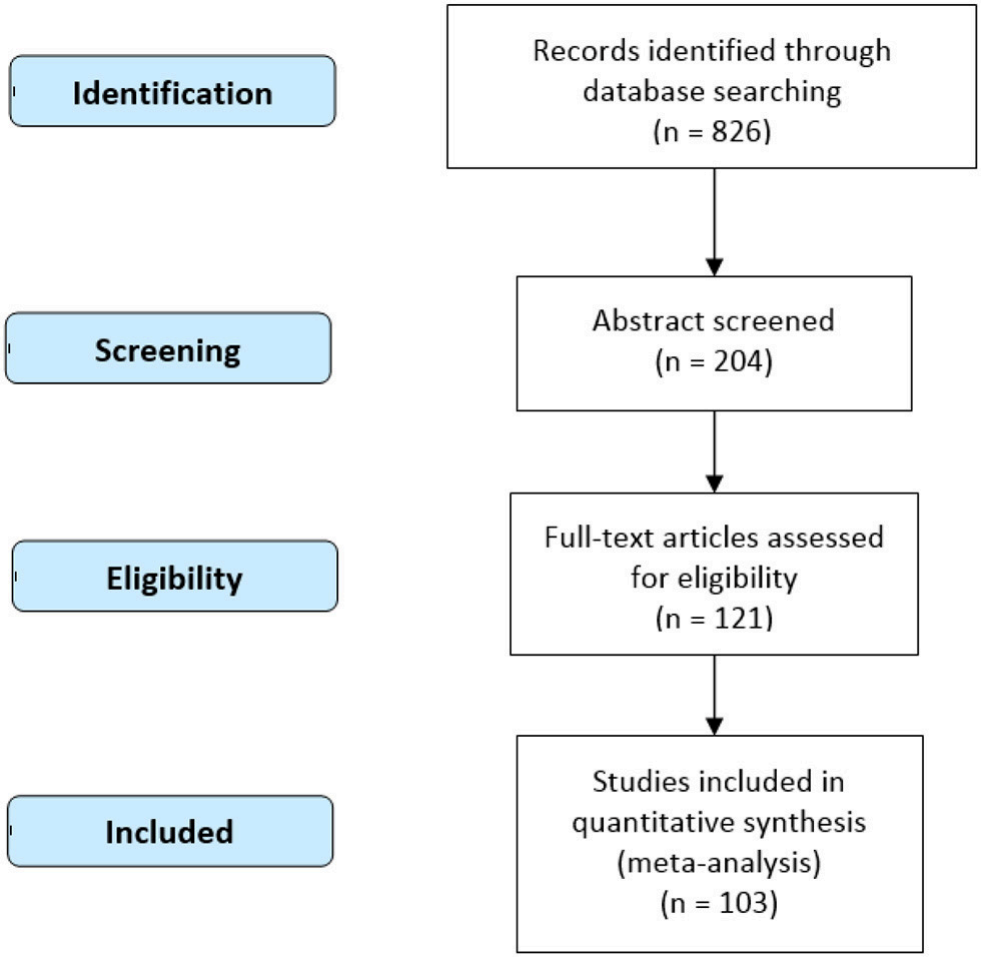
Procedure of data selection (PRISMA 2009 Flow Diagram).

Following this screening process, a total of 103 studies (Table 1 and Figure 2; see Supplementary Table 1 for PRISMA Checklist)) involving 2,085 participants (male = 1,116) and 2,586 foci were incorporated into the meta-analysis (see Supplementary Table 2). Among these foci, 1,372 were associated with large-scale spatial ability, while 1,214 were associated with small-scale spatial ability.

**Table 1:** Summary of studies included in the present meta-analysis.

**Study**	**N(Male)**	**Mean age**	**Country**	**Task and Contrast**	**Foci**
**Large-scale spatial ability**					
Janzen and Weststeijn (2007)	15(8)	22.6	Netherlands	Decision point items>non-decision point items in object location and route direction task, in-route items>against-route items	18
Nemmi et al. (2013)	19(11)	25.05	Italy	Route task>control	13
Ohnishi et al. (2006)	246(133)	30.2	Japan	maze task>control	22
Ino et al. (2002)	16(16)	32.3	Japan	Mental navigation task>control	10
Hartley and Maguire (2003)	16(16)	28.9	UK	Good navigation performance>poor navigation performance, wayfinding>trail following, wayfinding>route following	23
Baumann et al. (2010)	17(17)	31.6	Australia	Navigation task>control, good navigators>poor navigators	64
Janzen and Jansen (2010)	20(10)	24.75	Netherlands	Objects seen once at a decision point in wayfinding task>once at a non-decision point	7
Xu et al. (2010)	20(20)	24.2	Norway	The conditions of Normal, Without and Blocked in navigation task>Line following	83
Schinazi and Epstein (2010)	16(5)	23	USA	Decision point in real-world route learning>non-decision point, in-route>against-route	16
Latini-Corazzini et al. (2010)	16(16)	21.2	Italy	Route task>control, survey task>control, route task>survey task	31
Iaria et al. (2008)	10(5)	23.08	Canada	Highly familiar pathway in navigation task>control, bottom-up attention mechanisms>control, direct events>action events and perceptual event	86
Rosenbaum et al. (2004)	10(10)	26.4	Canadian	Mental navigation task>control	35
Rauchs et al. (2008)	16(8)	22.1	Canada	Common navigation task>control	62
Wolbers et al. (2007)	13(7)	21–29	USA	Path integration>control	16
Wolbers and Büchel (2005)	11(11)	19–28	Germany	Learning, performance, and change phase in navigation task>control	17
Hirshhorn et al. (2011)	13(5)	26.7	Unknown	Distance judgment, Proximity judgment, blocked route problem solving, and landmark sequencing>control	33
Rosenbaum et al. (2007)	7(4)	46.57	Canada	House recognition task>control	9
Brown et al. (2010)	20(9)	21.36	USA	Overlapping>non-overlapping	69
Iaria et al. (2007)	9(4)	24.9	Canada	Acquisition of the cognitive map and using the cognitive map>control	35
Grön et al. (2000)	24(12)	26	Germany	Navigation task>control	18
Weniger et al. (2010)	19(11)	25	Switzerland	Decide point during navigation in a virtual maze>control	17
Pine et al. (2002)	20(5)	13.9–28.8	USA	memory-guided navigation task and arrow- guided navigation task>control	27
Iaria et al. (2003)	14(7)	25.3	Canada	Place-learning task>control	17
Shelton and Gabrieli (2002)	12(6)	23.1	USA	Route encoding>fixation, survey encoding>fixation, route encoding>survey encoding	52
Maguire et al. (1997)	11(11)	45	UK	Routes, landmarks, film plots, and film frames tasks>control	35
IglóiI et al. (2010)	19(19)	24.3	UK	Training trials in navigation>control trials, allocentric and egocentric responses trials>control trials, allocentric responses trials>egocentric responses trials	95
Marsh et al. (2010)	25(21)	32.5	USA	Spatial learning>control	27
Rodriguez (2010)	11(5)	24–39	USA	Allocentric test>cue-place test, encoding>test phases	51
Orban et al. (2006)	24(12)	23.5	Belgium	Navigation task>control	24
Moffat et al. (2006)	30(15)	27	Unknown	Younger adults and older adults>control	46
Lambrey et al. (2012)	18(9)	21	UK	Self-rotation conditions in perspective taking task>table rotation conditions, self-rotation conditions>array rotation conditions, good performance>poor performance	13
Ino et al. (2007)	1(1)	55	Japan	Navigation task>control	7
Whittingstall et al. (2014)	18(5)	20–28	Canada	Visuospatial imagery task>control	14
Kaiser et al. (2008)	24(12)	28.15	Germany	Third-person-perspective>first-person-perspective	14
Hirshhorn et al. (2012)	16(6)	26.43	Canada	Navigation task>control	12
Pintzka et al. (2016)	53(0)	22.5	Norway	Successful>failed navigation	23
Jandl et al. (2015)	26(13)	63.7	Germany	Navigation trials>visual memory trials	12
Ledoux et al. (2013)	22(16)	30.48	Canada	Wayfinding task>control	13
Powell et al. (2012)	82(32)	21.6	UK	Landmark task>control	28
Ganesh et al. (2015)	23(11)	23	Netherlands	Egocentric mental spatial transformations>allocentric mental spatial transformations	8
Etchamendy et al. (2012)	30(12)	26.53	Canada	Concurrent spatial discrimination learning task>control	12
Clemente et al. (2013)	14(0)	21.64	Spain	Navigation>video, navigation>photographs	10
Mazzarella et al. (2013)	20(9)	27.7	Unknown	Altercentric condition in perspective taking task>control, egocentric condition>control	94
Lee et al. (2005)	10(10)	22–25	Hong Kong	Spatial orientation task>control	39
Lux et al. (2003)	14(14)	26.8	Germany	Spatial orientation task>control	10
Kesler et al. (2004)	13	14.5	USA	Spatial orientation task>control, difficult>control	8
**SMALL-SCALE SPATIAL ABILITY**					
Hugdahl et al. (2006)	11(6)	30	Norway	Mental rotation task>control	4
Schöning et al. (2007)	34(14)	32	Germany	Mental rotation task>control	95
Gogos et al. (2010)	10(0)	55.4	Australia	Mental rotation task>control	16
Weiss et al. (2009)	16(16)	20–39	Germany	Mental rotatio>stimulus categorization, mirrored presentation>non-mirrored presentation	59
Suchan et al. (2002)	10(4)	28.9	Germany	Mental rotation task>control	27
Wraga et al. (2010)	18(8)	22	USA	Perspective task>object-in-hand task, rotation task>fixation	50
Johnston et al. (2004)	9(5)	25.8	UK	Different orientation>same orientation	3
Suchan et al. (2006)	11(6)	27	Germany	Simultaneous matrix rotation>simultaneous 3-D cube rotation, simultaneous 3-D cube rotation>simultaneous matrix rotation, successive matrix rotation>successive 3-D cube rotation	19
Jordan et al. (2001)	9(1)	21	Germany	Three mental rotation conditions>control	36
Podzebenko et al. (2002)	10(5)	28.3	Australia	Mental rotation task>control	14
Seurinck et al. (2005)	24(0)	23	Belgium	Mental rotation task>control	36
Creem-Regehr et al. (2007)	16(7)	21	USA	Hand and viewer rotation tasks>control	35
Sluming et al. (2007)	10(10)	41	UK	Mental rotation task>control	14
Wolbers et al. (2003)	13(9)	Unknown	Germany	Mental rotation task>control	11
Keehner et al. (2006)	14(7)	Unknown	Australia	Different degrees of rotation>control	10
Lamm et al. (2007)	13(13)	23–31	Austria	Location and orientation condition during mental rotation>control	16
Vingerhoets et al. (2002)	13(13)	29	Belgium	Rotated hands and figures>control	31
Wraga et al. (2005)	11(7)	25	USA	Object rotation task>control, self-rotation task>control	51
Corradi-Dell'Acqua et al. (2009)	17(17)	28.31	Germany	Body schema and body structural rotation>control, stimulus strategy>control	5
Halari et al. (2006)	19(9)	25.78	UK	Mental rotation task>control	27
Creem et al. (2001)	12(6)	24	USA	Mental rotation task>control	15
Thomsen et al. (2000)	11(6)	30	Norway	Mental rotation task>control	4
Vingerhoets et al. (2001)	10(5)	26	Belgium	Mental rotation task>control	5
de Lange et al. (2005)	6(6)	25	Netherlands	Mental rotation task>control	10
Logie et al. (2011)	21(7)	30–35	UK	Mental rotation>control, high imagers>low imagers	16
Levin et al. (2005)	12(6)	20.67	USA	Mental rotation task>control	18
De Lange et al. (2006)	17(17)	24	Netherlands	mental rotation task>control	7
Ferri et al. (2014)	18(9)	24	Italy	Mental rotation of one's own right hand>mental rotation of one's own left and other's hand, angular orientation>control	25
Kawamichi et al. (2007)	14(14)	18–33	Japan	Mental rotation task>control	40
Jordan et al. (2002)	24(10)	23.17	Germany	Three mental rotation conditions (3d, abstract, letter)>control, 3D-condition>the ABSTRACT- and LETTER-conditions	36
Lamm et al. (2001)	13(13)	24.5	Austria	Mental rotation task>control	11
Paschke et al. (2012)	10(10)	25	Germany	mental rotation task>control	3
Milivojevic et al. (2008)	14(8)	26.21	New Zealand	Stimulus rotation during a mirror-normal parity judgment task>letter–digit category judgment task	15
Ng et al. (2001)	12(12)	29.25	UK	Line orientation experiment>control, mental rotation experiment>control	15
Stoodley et al. (2012)	9(9)	25	USA	Rotated letters>upright letters	18
Wilson and Farah (2006)	7(3)	18–23	USA	Letter and object mental rotation>control	11
Papeo et al. (2012)	18(0)	22–28	USA	Motor strategy and visuospatial strategy>control	15
Schendan and Stern (2007)	16(7)	21.2	USA	Mental rotation task>control	26
Kucian et al. (2006)	20(10)	27.2	Switzerland	Mental rotation task>control	13
Zacks et al. (2002)	24(9)	19–31	USA	Spatial transformation tasks>control	36
Seurinck et al. (2011)	16(16)	24	Netherlands	Mental rotation task>control	16
Seurinck et al. (2004)	22(11)	25.4	Belgium	Mental rotation task>control	46
Vanrie et al. (2002)	6(3)	25.2	Belgium	Mental rotation task>control	60
Carrillo et al. (2010)	42(23)	31.89	Spain	Mental rotation task>control	2
O'Boyle et al. (2005)	6(6)	14.3	Australia	Mental rotation task>control	7
Ecker et al. (2006)	10(0)	20–30	UK	Mental rotation task>control	15
Baumann et al. (2012)	14(7)	21.5	Australia	Spatial relations task>control	4
Bodin et al. (2010)	11(5)	20–35	Sweden	Mental rotation task>control	20
Gao et al. (2017)	30(15)	19–25	China	Mental rotation task>control	10
Prescott et al. (2010)	8(8)	14.2	Australia	Mental rotation task>control	18
Goh et al. (2013)	97(50)	22.6	USA	Visuo-spatial judgments>control	7
Wolbers et al. (2006)	16(16)	19–29	Germany	Spatial visualization task>control	6
Ebisch et al. (2012)	22(0)	20–24	Italy	Induction–visualization>induction–spatial relationships, visualization–induction>visualization–spatial relationships	8
Blacker and Courtney (2016)	32(8)	18–30	USA	Spatial relations>location	36
Newman et al. (2016)	36(21)	8	USA	Mental rotation task>control	8
Kucian et al. (2005)	22(10)	25.9	Switzerland	Mental rotation task>control	37
Elizabeth and Paul (2011)	16(4)	18–28	USA	Matching in embedded figures task>searching	16

### Activation Likelihood Estimation (ALE)

ALE, which treats activation foci reported in neuroimaging studies not as single points but as spatial probability distributions centered at the given coordinates, is the most common algorithm for coordinate-based meta-analysis (Eickhoff et al., 2012). In the present study, we utilized the revised algorithm for ALE analysis proposed by Eickhoff et al. (2009). This algorithm models the spatial uncertainty—and thus the probability distribution—of each focus using an estimation of the inter-individual and inter-laboratory variability typically observed in neuroimaging experiments, rather than using a pre-specified full-width at half maximum (FWHM) for all experiments as originally proposed. The modified permutation procedure reflects the null-distribution of a random spatial association between studies (i.e., random-effects analysis), rather than between foci (i.e., fixed-effects analysis; Eickhoff et al., 2016, 2017). The “modeled activation” (MA) map is computed by the following procedure. First, all foci reported for a given study are modeled as Gaussian probability distributions. The information provided by the foci of a given study is then merged into a single 3D-volume. To this end, the modeled probabilities are combined over all foci reported in that experiment by taking the voxel-wise union of their probability values (Eickhoff et al., 2009). The respective activation probabilities (the values of the MA maps) are then recorded, yielding as many values as there had been studies included in the current meta-analysis. These values correspond to MA values, that were sampled from random, spatially independent locations. The union of these activation probabilities is then computed in the same manner as done for the meta-analysis itself in order to yield an ALE score under the null-hypothesis of spatial independence (Eickhoff et al., 2009, 2012).

The ALE method is advantageous in that it may help to resolve several issues in current brain imaging research. First, the number of participants involved in single brain imaging studies is generally low, often providing unstable results. Second, single-brain imaging results are likely influenced by certain experimental conditions (e.g., scan parameters). Third, interpretations regarding the function of a given brain region derived from a single study are often based on a small number of experimental tasks.

Ginger ALE software (version 2.3; http://www.brainmap.org/ale) was used to conduct the current meta-analysis, including the conversion of Talairach coordinates into MNI space. Loci in both cerebral and cerebellar regions were all focused by the current research. The resulting *p*-value maps were threshold using cluster-level family-wise error (FWE) correction at *p* < 0.05, with a cluster-defining threshold of *p* < 0.005 and 5,000 permutations (Eickhoff et al., 2012). The results were overlaid onto an anatomical template (Colin27 T1 seg MNI.nii; http://www.brainmap.org/ale) and displayed using Mango software (http://rii.uthscsa.edu/mango).

## Results

**Figure 3:**
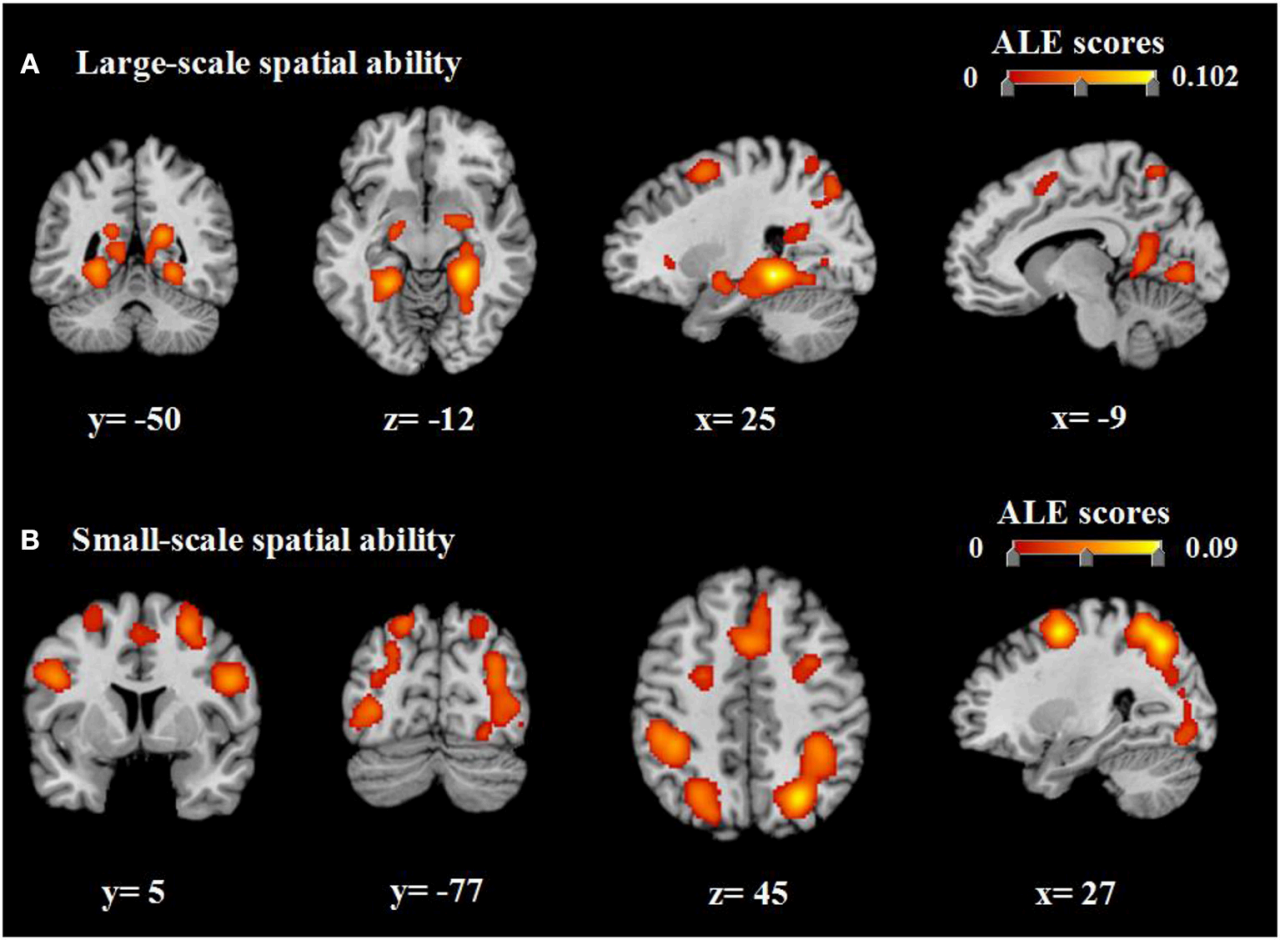
ALE meta-analysis of neuroimaging studies regarding large-scale spatial ability **(A)** and small-scale spatial ability **(B)**. Coordinates are presented in millimeters (mm). ALE, activation likelihood estimation.

Our ALE analysis identified 16 clusters of consistent activation related to large-scale spatial ability. The largest two clusters of activation in the bilateral limbic lobe were centered in the bilateral parahippocampal gyrus. Other clusters were primarily located in the bilateral posterior lobe, bilateral occipital lobe, bilateral parietal lobe, right anterior lobe, bilateral frontal lobe, and right sub-lobar area (Table 2, Figure 3). Clusters exhibiting consistent activation in the analysis of small-scale spatial ability were concentrated mainly within the bilateral parietal lobe, bilateral occipital lobe, bilateral frontal lobe, right posterior lobe, and left sub-lobar area (Table 3 and Figure 3).

**Table 2:** ALE meta-analysis results for large-scale spatial ability.

**Cluster No**.	**Hemisphere**	**Brain regions**	**BA**	**x**	**y**	**z**	**ALE(10^**−2**^)**	**Cluster size (mm3)**
1	R	Parahippocampal gyrus	36	26	−38	−10	0.102188	9912
	R	Declive		26	−60	−12	0.032387	
	R	Parahippocampal gyrus		36	−22	−22	0.026213	
2	L	Parahippocampal gyrus	36	−24	−46	−6	0.079631	5784
3	R	Superior occipital gyrus	19	42	−78	32	0.051903	4400
	R	Middle occipital gyrus	19	40	−80	22	0.046983	
	R	Precuneus	7	26	−76	46	0.034177	
	R	Precuneus	7	26	−66	36	0.024326	
4	L	Posterior cingulate	30	−16	−56	18	0.07205	4224
5	R	Posterior cingulate	30	18	−52	16	0.072787	3512
	R	Culmen		10	−46	2	0.037102	
6	L	Lingual gyrus	18	−6	−80	−2	0.050804	3384
	R	Lingual gyrus	18	18	−68	0	0.036607	
	R	Culmen		6	−72	−2	0.034144	
	L	Declive		−16	−76	−8	0.027501	
7	R	Sub-gyral	6	28	6	54	0.049654	2856
8	R	Precuneus	7	18	−62	58	0.048159	2752
	L	Precuneus	7	−2	−66	54	0.031828	
9	R	Superior frontal gyrus	6	4	14	50	0.04669	2096
10	L	Middle occipital gyrus	19	−32	−86	24	0.057178	1896
	L	Superior occipital gyrus	19	−32	−84	38	0.025026	
	L	Cuneus	17	−20	−80	20	0.02431	
11	L	Superior parietal lobule	7	−18	−62	62	0.044397	1832
12	L	Sub-gyral	6	−26	0	58	0.048783	1752
13	L	Inferior occipital gyrus	19	−42	−78	0	0.043239	1504
	L	Middle occipital gyrus	19	−44	−82	12	0.025768	
14	R	Lentiform nucleus		18	−4	−12	0.0353	1376
	R	lentiform nucleus		26	−6	−12	0.034406	
	R	Parahippocampal gyrus		22	−10	−16	0.031555	
15	L	Parahippocampal gyrus		−26	−20	−18	0.028272	1256
16	R	Claustrum		32	24	−4	0.052402	1112

*BA, Brodmann area; R, right; L, left; ALE, activation likelihood estimation*.

**Table 3:** ALE meta-analysis results for small-scale spatial ability.

**Cluster No**.	**Hemisphere**	**Brain regions**	**BA**	**x**	**y**	**z**	**ALE(10^**−**^^**2**^)**	**Cluster size (mm3)**
1	R	Precuneus	7	26	−66	48	0.089947	24816
	R	Precuneus	7	26	−56	56	0.075651	
	R	Precuneus	31	34	−74	26	0.049883	
	R	Inferior parietal lobule	40	36	−40	42	0.048361	
	R	Middle occipital gyrus	18	34	−82	2	0.042117	
2	L	Superior parietal lobule	7	−26	−64	50	0.059831	22584
	L	Inferior parietal lobule	40	−38	−40	46	0.056222	
	L	Inferior parietal lobule	40	−46	−36	46	0.048675	
	L	Precuneus	7	−16	−62	54	0.047647	
	L	Precuneus	7	−20	−74	48	0.045286	
	L	Inferior occipital gyrus	19	−42	−72	−4	0.043441	
	L	Inferior occipital gyrus	19	−36	−78	0	0.041091	
	L	Middle occipital gyrus	18	−30	−90	12	0.039092	
	L	Inferior occipital gyrus	18	−36	−86	−2	0.037764	
	L	Middle occipital gyrus	19	−34	−78	18	0.024928	
	L	Precuneus	31	−26	−78	24	0.024692	
3	R	Middle frontal gyrus	6	28	−2	56	0.086822	4968
4	L	Middle frontal gyrus	6	−24	−4	54	0.073822	4880
5	R	Inferior frontal gyrus	9	52	8	26	0.070489	4696
6	L	Superior frontal gyrus	6	2	12	50	0.072562	4608
	R	Medial frontal gyrus	8	6	26	46	0.030976	
7	L	Inferior frontal gyrus	9	−48	8	28	0.057083	3160
8	R	Tuber		42	−62	−32	0.040321	2944
	R	Declive		44	−64	−16	0.036898	
9	L	Insula	13	−32	26	0	0.039269	976

**Figure 4:**
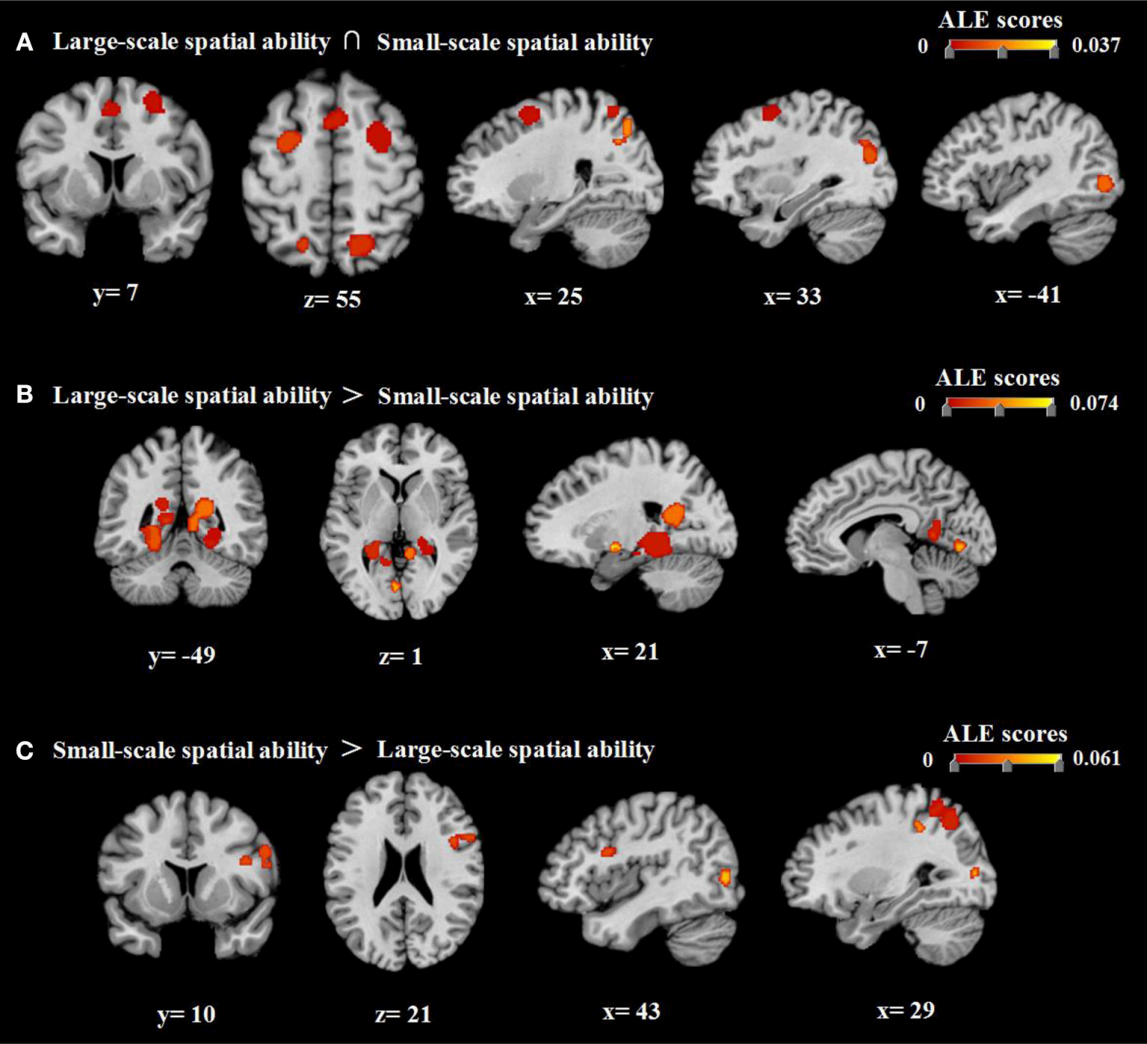
Results of conjunction and contrast analyses. **(A)** The common regions associated with large- and small-scale spatial ability. **(B)** Brain regions exhibiting greater activation for large-scale spatial ability than for small-scale spatial ability. **(C)** Brain regions exhibiting greater activation for small-scale spatial ability than for large-scale spatial ability. Coordinates are presented in millimeters (mm).

We then compared the ALE results for large- and small-scale spatial abilities to identify common and distinct neural regions associated with each ability. Conjunction analysis revealed that bilateral sub-gyrus, right superior frontal gyrus (SFG), right superior parietal lobule (SPL), right middle occipital gyrus (MOG), right superior occipital gyrus (SOG), left inferior occipital gyrus (IOG), and bilateral precuneus were activated for both large- and small-scale spatial abilities (Table 4 and Figure 4). In addition, large- and small-scale spatial ability contrasts demonstrated correspondence in the bilateral parahippocampal gyrus, left lingual gyrus, bilateral culmen, right middle temporal gyrus (MTG), left declive, left SOG, and right lentiform nucleus (Table 5 and Figure 4). Conversely, small- and large-scale spatial ability contrasts converged in the bilateral precuneus, right inferior frontal gyrus (IFG), right precentral gyrus, left inferior parietal lobule (IPL), left supramarginal gyrus, left SPL, right IOG, and left middle frontal gyrus (MFG; Table 5 and Figure 4).

**Table 4:** Results of conjunction analysis for large- and small-scale spatial ability.

**Cluster No**.	**Hemisphere**	**Brain regions**	**BA**	**x**	**y**	**z**	**ALE(10^**−2**^)**	**Cluster size (mm3)**
1	R	Sub-Gyral	6	26	4	56	0.049543	1760
2	R	Superior frontal gyrus	6	4	14	50	0.04669	1704
3	R	Superior parietal lobule	7	20	−62	58	0.045159	1616
4	L	Sub-Gyral	6	−26	0	58	0.048783	1440
5	R	Middle occipital gyrus	19	34	−78	20	0.035724	1136
	R	Superior occipital gyrus	19	36	−74	30	0.03098	
6	L	Inferior occipital gyrus	19	−42	−76	−2	0.036802	664
7	L	Precuneus	7	−18	−62	58	0.036471	640
8	R	Precuneus	7	26	−74	48	0.032807	464
9	R	Precuneus	7	26	−66	36	0.024326	120

**Table 5:** Results of contrast analysis for large- and small-scale spatial ability.

**Cluster No**.	**Hemisphere**	**Brain regions**	**BA**	**X**	**Y**	**Z**	**ALE(10^**−2**^)**	**Cluster size (mm3)**
**LARGE-SCALE SPATIAL ABILITY>SMALL-SCALE SPATIAL ABILITY**								
1	R	Parahippocampal gyrus	35	25	−36.8	−10.5	0.09013	6368
	R	parahippocampal gyrus	35	32	−23.3	−22	0.021	
2	L	Lingual gyrus		−14.7	−56.5	14.6	0.05872	4016
3	L	Culmen		−24.3	−43	−7.5	0.05677	3536
	L	Culmen		−20.3	−42.3	−17.7	0.02638	
4	R	Culmen		17.4	−52.2	14.6	0.069	3392
5	R	Middle temporal gyrus	19	41	−76	35.4	0.0448	936
6	L	Culmen	0	−3.5	−73.2	−1.7	0.0239	616
	L	lingual gyrus	18	−10	−76	−6	0.02136	
	L	Declive		−14.7	−77.3	−8.7	0.02416	
7	L	Superior occipital gyrus	19	−36.2	−83.8	30	0.03538	464
8	R	Lentiform nucleus		15	−2.7	−12.2	0.0278	440
	R	Lentiform nucleus		20	−3	−10	0.02655	
	R	Lentiform nucleus		22	−8	−10	0.02424	
9	L	Parahippocampal gyrus		−28	−24	−16	0.02198	128
	L	Parahippocampal gyrus		−26	−18	−17	0.00386	
**SMALL-SCALE SPATIAL ABILITY>LARGE-SCALE SPATIAL ABILITY**								
1	R	Precuneus	7	26.6	−63.3	48.7	0.0659	3008
	R	Precuneus	7	25.3	−54.7	59	0.05862	
2	R	Inferior frontal gyrus	9	55.1	10.9	28.7	0.03762	1352
	R	Precentral gyrus	6	58	8	30	0.02762	
	R	inferior frontal gyrus	44	55	12	19	0.02476	
	R	Precentral gyrus	6	43	9.3	21.3	0.02805	
	R	Precentral gyrus	6	44	4	22	0.01912	
	R	Inferior frontal gyrus	9	47	14	20	0.02064	
3	L	Inferior parietal lobule	40	−48	−40	46	0.03136	968
	L	Inferior parietal lobule	40	−47	−43	49	0.02126	
	L	Supramarginal gyrus	40	−46.7	−36.7	41.3	0.0275	
	L	Inferior parietal lobule	40	−38	−48	58.5	0.02253	
4	L	Superior parietal lobule	7	−25	−74	52	0.0222	936
	L	Precuneus	7	−21.7	−61.4	45.7	0.02749	
	L	Precuneus	7	−23	−53	55	0.02721	
5	R	Inferior occipital gyrus	19	37.5	−79.4	6.2	0.03082	888
6	L	Middle frontal gyrus	6	−20.3	−6.6	52.9	0.0387	568
	L	Middle frontal gyrus	6	−22	−9.2	62	0.02362	
7	R	Precuneus	7	30	−42	42	0.025	504

## Discussion

In the present study, we conducted an ALE meta-analysis to elucidate the neural basis of large- and small-scale spatial abilities. Our results indicated that large-scale spatial tasks were associated with increased activation in the parahippocampal gyrus, SOG, MOG, precuneus, posterior cingulate, lingual gyrus, sub-gyrus, right SFG, left cuneus, and left SPL. This result is consistent with the findings of Boccia et al. (2014) and Kühn and Gallinat (2014) regarding individual navigational capabilities. Furthermore, cognitive processing of individual large-scale spatial information was associated with activation in the declive, culmen, left IOG, right lentiform nucleus, and right claustrum. These findings are in accordance with those of several previous studies, which have also suggested that the parahippocampal gyrus plays a key role in large-scale spatial ability (Connor and Knierim, 2017; Cullen and Taube, 2017; Epstein et al., 2017; Lisman et al., 2017; Moser et al., 2017).

Our analysis also revealed that small-scale spatial tasks were associated with higher activation in the IPL, MOG, left SPL, left IOG, MFG, and IFG. These results are consistent with the findings of Tomasino and Gremese (2016) regarding individual mental rotation capabilities. Furthermore, cognitive processing of individual small-scale spatial information was also associated with activation in the precuneus, right MFG, right tuber, right declive, and left insula.

Notably, our ALE conjunction analysis revealed that the sub-gyrus, right SFG, right SPL, right MOG, right SOG, left IOG, and precuneus participate in the cognitive processing of both large- and small-scale spatial information. These results are in accordance with the model proposed by Hegarty et al. (2006), which argues that there is a partial intersection/association between individual large-scale and small-scale spatial abilities. Hegarty et al. (2006) speculated that this association arises from the fact that spatial ability is not a completely independent cognitive function, as both large- and small-scale spatial ability involve cognitive processes such as visual processing and working memory. Indeed, our results support this notion, suggesting a shared neural basis for large- and small-scale spatial ability. Extensive research has demonstrated that the sub-gyrus, right SPL, right MOG, right SOG, left IOG, and precuneus are associated with the processing of visual spatial information (Fink et al., 2003; Price and Devlin, 2003; Bristow et al., 2005; Konen et al., 2005; Slotnick and Moo, 2006; Deutschländer et al., 2008; Choi et al., 2010; Seo et al., 2012). Additional studies have indicated that the sub-gyrus, right SFG, and precuneus are significantly associated with working memory tasks (Mitchell, 2007; Chein et al., 2011; Fassbender et al., 2011; Ma et al., 2012; Opitz et al., 2014). Furthermore, the right sub-gyrus, right SFG, right MOG, and precuneus have been associated with attention (Wu et al., 2007; Kelley et al., 2008; Thakral and Slotnick, 2009; Li et al., 2012; Majerus et al., 2012), while the sub-gyrus and left precuneus have been associated with the planning process (Wagner et al., 2006; Jankowski et al., 2009; Crescentini et al., 2012). The sub-gyrus has also been implicated in executive function (Kerstin et al., 2005; Schubotz and von Cramon, 2009; Jin et al., 2012; Dambacher et al., 2014).

Taken together, these findings suggest that both large- and small-scale spatial ability involve visual processing and working memory of spatial information, as well as cognitive processes such as attention, planning, and executive control. These processes may represent vital cognitive and behavioral foundations for the overlap and interaction between large- and small-scale spatial ability. In this context, an individual first relies on attention to capture visual spatial information, following which working memory aids in the preparation and processing of the input. The individual then develops a detailed plan to finish the current spatial cognitive task. The planning process is often embodied in the corresponding task execution strategy for large-scale spatial tasks. For small-scale spatial tasks, it is often embodied in the operational scheme developed by the participant according to his/her own operational capabilities. Execution of the plan and behavioral responses during the experimental task are under the control of executive function.

We further analyzed the specific neural activity involved in large- and small-scale spatial abilities. Our analysis indicated that large-scale spatial tasks were associated with more intense activation of the parahippocampal gyrus, left lingual gyrus, culmen, right MTG, left declive, left SOG, and right lentiform nucleus. Indeed, previous studies have indicated that the parahippocampal gyrus, left lingual gyrus, culmen, and right MTG are significantly associated with the processing of information related to virtual environments (Weniger et al., 2009, 2013; Mellet et al., 2010; Nieuwland, 2012; Fairhurst et al., 2013). Additional studies have demonstrated that the parahippocampal gyrus, left lingual gyrus, culmen, and left SOG are associated with the processing of information related to construction, scene, and place (Suzuki et al., 2005; Henderson et al., 2008; Mullally et al., 2012; Rémy, 2014; Sulpizio et al., 2014; Zeidman et al., 2015). The parahippocampal gyrus, left lingual gyrus, and culmen have also been implicated in autobiographical memory (Maguire and Dhassabis, 2010; Whalley et al., 2012; Wilbers et al., 2012). Several studies have further reported that the parahippocampal gyrus, culmen, and left SOG are associated with episodic memory (Suzuki et al., 2009; Reggev et al., 2011; Demaster and Ghetti, 2013), while the right parahippocampal gyrus, left lingual gyrus, left culmen, and left declive are associated with semantic memory (Grossman et al., 2003; Assaf et al., 2005; Kodama et al., 2015). Recent research has also demonstrated that the right lentiform nucleus is associated with emotional processing (Goldin et al., 2008; Telzer et al., 2014; Wardle et al., 2014; Touroutoglou et al., 2015). Taken together, these findings suggest that, relative to small-scale spatial ability, large-scale spatial ability relies more heavily on the cognitive processing of spatial information related to aspects of the virtual environment (e.g., construction, scene, place, etc.), and on the autobiographical, episodic, and semantic aspects of memory. Our findings further suggest that large-scale spatial ability is associated with greater emotional responses.

In contrast, small-scale spatial tasks were associated with more intense activation in the precuneus, right IFG, right precentral gyrus, left IPL, left supramarginal gyrus, left SPL, right IOG, and left MFG. Previous studies have revealed that the precuneus, right IFG, right precentral gyrus, left IPL, left SPL, right IOG, and left MFG are associated with motor imagery (Kaladjian et al., 2007; De Lange, 2008; Chen et al., 2009; Thompson and Baccus, 2012; Chinier et al., 2014; Lissek et al., 2014). Additional studies have demonstrated that the precuneus, right IFG, right precentral gyrus, left supramarginal gyrus, left IPL, and left MFG are associated with control of the finger and hand, particular for grasping and tapping movements (Culham et al., 2003; Vandermeeren et al., 2003; Müller et al., 2011; Schweisfurth et al., 2011; Akhlaghi et al., 2012; Lavrysen et al., 2012; Stefanics and Czigler, 2012). Moreover, the right precentral gyrus, left IPL, and left supramarginal gyrus have been associated with working memory (Mitchell, 2007; Burgess and Braver, 2010; Chein et al., 2011; Fassbender et al., 2011). In addition, several studies have indicated that the precuneus, right IFG, right precentral gyrus, left IPL, and left supramarginal gyrus are involved in arithmetic and calculation (Gruber et al., 2001; Fehr et al., 2007; Benn et al., 2013). Some studies have also reported that the right precentral gyrus and right IFG are assocaited with matching activities (Darcy Burgund et al., 2005; Soulières et al., 2009). Taken together, these findings indicate that, relative to large-scale spatial ability, small-scale spatial ability relies more heavily on motor imagery, mental processes associated with finger and hand movements, and cognitive processes such as working memory, calculation, and matching.

However, it is noteworthy that although there are brain regions related to individuals' working memory in the common part of large- and small-scale spatial ability and in the unique part of the small-scale spatial ability, these brain regions are still different with respect to the cognitive process and the functions they are specifically involved in. First of all, the cognitive process shows that the working memory includes such three processes as encoding, maintenance, and retrieval; however, the sub-gyrus in the common part is involved in the encoding process, precuneus is involved in the maintenance process, and SFG is involved in the retrieval process; whereas the precentral gyrus in the unique part is involved in the encoding and maintenance processes, IPL is involved in the retrieval process, and supramarginal gyrus is involved in the encoding and retrieval processes (Langel et al., 2014). Then, the function of the working memory shows that these brain regions serve as the significant neural foundations of the central executive system in individuals' working memory. The central execution system, as the core component of the working memory, has such important functions as selection, inhibition, updating, and conversion (Collette and Linden, 2002). In the common part, however, SFG is mainly responsible for updating in the central executive system, and precuneus is mainly responsible for selection. In addition, IPL and supramarginal gyrus in the unique part are also crucial brain regions for updating (Bledowski et al., 2009; Borst and Anderson, 2013). To sum up, we believe that although both large- and small-scale spatial ability involves the cognitive process of encoding, maintenance, and retrieval in individuals' working memory, small-scale spatial ability also involves working memory processes that more precuneus, IPL and supramarginal gyrus are involved in. It can also be understood this way: although working memory is involved in the cognitive processing of large- and small-scale spatial information of individuals at the same time, the involvement in the latter is wider and deeper. On the other hand, the selection and updating functions of the central executive system in the working memory also constitute the common basis of individuals' large- and small-scale spatial ability, but the small-scale spatial ability, compared with the large-scale spatial ability, is more dependent on the updating function. Therefore, the result that this working memory is present in the common part of the large- and small-scale spatial ability and in the unique part of the small-scale spatial ability is not contradictory. Moreover, this result, to a certain extent, also mutually supports the phenomenon that the working memory training as found by many researchers in the domain of cognitive training can lead to a significant transfer influence on individuals' spatial ability (Alloway et al., 2013; Borella et al., 2017; Nilsson et al., 2017).

Although large- and small-scale spatial ability are to some extent associated at both the neural and behavioral levels, they are profoundly different in many ways. Large-scale spatial ability reflects more of the individual's ability to process spatial information related to actual or virtual situations. In such cases, regions associated with the cognitive processing of environmental information (e.g., construction, scene, place, etc.) will be activated more intensely, following which brain regions corresponding to autobiographical and episodic memory will be engaged. In addition, as large-scale spatial tasks often require more detailed explanation, semantic memory load will be greater for such tasks than for those associated with small-scale spatial ability. Interestingly, we also observed activation of emotion-related regions during large-scale spatial tasks. One explanation for this finding was that experimental tasks in general might elicit emotional responses. Individuals are more prone to such negative emotions as anxiety and fear when completing large-scale spatial tasks. For instance, Lawton (1994) and Malinowski and Gillespie (2001) found that wayfinding performance was negatively correlated with anxiety. Lawton and Kallai (2002) further discovered that women showed greater anxiety than men in large-scale spatial tasks. In addition, many scholars focus on the stress responses of individuals in navigation tasks (Duncko et al., 2007; Schwabe et al., 2007, 2009). Therefore, when compared with small-scale spatial ability, the large-scale spatial ability of individuals is more often studied by being linked to emotional factors. Consistent with these studies, our findings also showed that individuals have actually activated the emotion-related brain regions when completing large-scale spatial tasks. Alternatively, experimental materials depicting beautiful scenery, architecture, or familiar scenes may evoke various emotional responses among individual participants. Such task elements as the stimulus materials and the experimental scenarios for the large-scale spatial tasks at this stage are much more plentiful than smaller-scale space tasks. It is for sure that this also prompts them to have more elements affecting individual emotions potentially. So, can the improved and enriched small-scale spatial tasks or research paradigms affect individuals' activities and emotion-related brain regions? We believe that this question still warrants validation of more studies.

**Figure 5:**
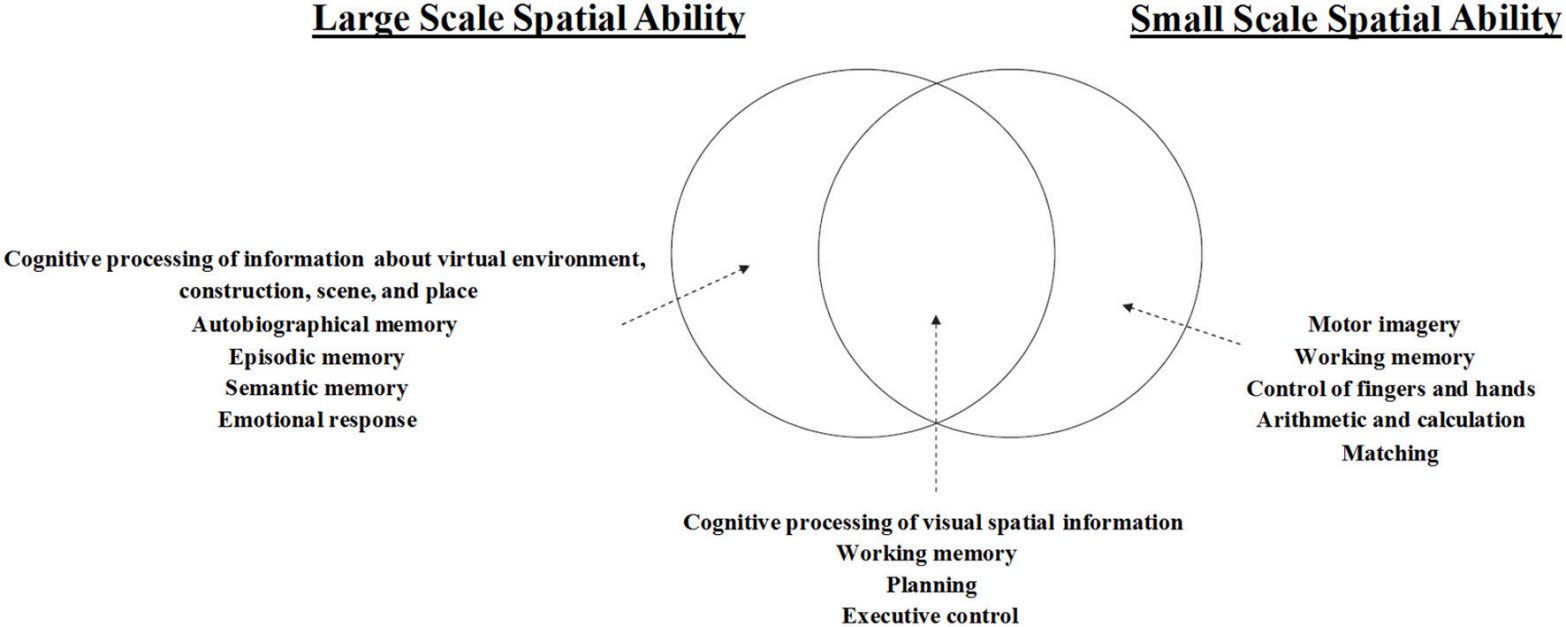
Model of the relationship between large- and small-scale spatial ability based on the current findings.

In contrast, our findings indicated that small-scale spatial ability was more strongly associated with brain regions involved in motor imagery, grasping, calculation, and matching, suggesting that this form of spatial ability reflects an individual's ability to process spatial information for specific objects. In this case, judgments are made based on imagination and mental representations. Thus, grasping, calculation, and matching functions may aid individuals in operating upon mental representations. Because imagination and operation abilities reflect working memory function, our findings suggest that small-scale spatial ability relies more heavily on working memory than other aspects of memory function. Given these findings, we propose a new model, as shown in Figure 5.

The current model was proposed on basis of Hegarty et al. (2006)'s model. We validated and supplemented their model from the neuroimaging level. We know that each region of our brain has many corresponding functions, and we proposed the current model mainly based on the following three considerations: first, based on our own literature accumulation and experience and taking into account the functional intelligence of the relevant brain regions from such database as Neurosynth (http://neurosynth.org/) and Brainnetome (http://atlas.brainnetome.org/bnatlas.html), we determined the aspects of cognitive function that brain regions mainly involve in the current ALE results; then, we examined whether these functions were consistent or directly associated with the cognitive activity components of the various components of Hegarty et al. (2006)'s model, thereby determining whether our findings can support Hegarty et al. (2006)'s model; lastly, by summarizing the work in the previous two steps, findings of previous studies, and our understanding of the individual spatial ability, we proposed the current model that would supplement and deepen Hegarty et al. (2006)'s model finally.

In general, the findings of the present research supported and extended the model proposed by Hegarty et al. (2006). With regard to the large-scale spatial ability, first, Hegarty et al. summed up the cognitive components specific to individuals' large-scale spatial ability based on his own studies, including addressing and spatial learning based on sensing of body motions. In fact, both “updating” and “learning” refer to the process of encoding the spatial stimulus information by individuals, and this spatial stimulus information is on basis of sensing of body motions. According to Hegarty et al. (2006), on the one hand, these stimuli were derived from the real environment; on the other hand, these stimulus messages also contained information regarding the “self.” Our model based on the current ALE results also supported such a view. According to our model, individuals' large-scale spatial ability not only involves individuals' processing of such real-world spatial stimulus information as constructions, scenes and places, but also contains individuals' cognitive processing of the virtual context space stimulus information. Moreover, we have also found that the brain regions associated with individuals' autobiographical memory, episodic memory, semantic memory and emotional responses are also deeply involved in individuals' large-scale spatial cognition, among which the first two items are associated with individuals' “self” information. Secondly, Hegarty et al. (2006) also thought that individuals' large- and small-scale spatial ability involves individuals' encoding of the visual spatial stimulus information, maintenance of spatial representations in the working memory and process of inference on the basis of these spatial representations. Not only does our model support the above conclusions fully, but also we further propose that all the processes of encoding, maintenance and retrieval, and the core central execution system components in the working memory are important for individuals' spatial ability. In addition, we believe that the final inference process involves mainly the ability of individuals' planning and executive control. Finally, as mentioned earlier (also as Figure 5), another important addition and extension of the current research to Hegarty et al. (2006)'s model was that we attempted to present the cognitive ability base specific to individuals' small-scale spatial ability.

Notably, we observed no distinct differences in the cognitive processing strategies utilized during large- and small-scale spatial tasks. Currently, strategies for processing spatial information are divided into three main categories: (1) egocentric-allocentric (Galati et al., 2000), (2) whole-part (Li and O'Boyle, 2011; Janssen and Geiser, 2012), and (3) motor imagination-visual processing (Tomasino and Gremese, 2016). However, the basis and theory of these three categories are quite similar, and we speculate that the latter two represent variations upon the first category. Whole-part strategies are likely to be utilized during the processing of large-scale spatial information, while motor imagination-visual processing strategies are likely to be utilized during the processing of small-scale spatial information. Some researchers have hypothesized that large-scale spatial tasks primarily require egocentric strategies, while small-scale tasks primarily require allocentric strategies (Malinowski, 2001; Zacks et al., 2001; Peña et al., 2008).

A meta-analysis by Boccia et al. (2014) revealed that navigation tasks were associated with greater activation of the right SOG, right angular gyrus, and right precuneus when egocentric rather than allocentric strategies are adopted. A subsequent meta-analysis by Tomasino and Gremese (2016) revealed that small-scale spatial tasks were associated with greater activation in the right postcentral gyrus, left IPL, left postcentral gyrus, left SPL, and right angular gyrus when egocentric strategies are adopted. In contrast, allocentric strategies were associated with greater activation of the right precuneus, right SFG, right SOG, left MOG, left SPL, left inferior temporal gyrus (ITG), left IOG, left MOG, and right posterior medial frontal gyrus. Interestingly, the right precuneus, right SOG, and left SPL reflect opposing processing strategies for large- and small-scale spatial tasks. Taken together, these findings suggest that there is no absolute difference in cognitive processing strategies between large- and small-scale spatial ability, and that either egocentric or allocentric strategies may be utilized in both task types. Differences in strategy selection may be related to individual differences such as the interaction between gender and verbal working memory (VWM)/visual spatial working memory (VSWM) ratio (Wang and Carr, 2014).

The present study possesses several limitations of note. First, unlike meta-analyses performed in other fields of research, ALE calculations based on neuroimaging do not consider the size of an effect; consequently, they cannot include evidence for the absence of an effect (i.e., so-called null results). Furthermore, ALE analyses cannot illuminate the temporal dynamics of cognitive processes (Winlove et al., 2018). Secondly, as most participants in the included studies were relatively young, we were unable to explore the effect of life-long development on the relationship between large- and small-scale spatial ability. Finally, we investigated spatial ability in healthy individuals only. Further studies are required to determine whether similar results can be obtained in patients with major depressive disorder, schizophrenia, attention deficit hyperactivity disorder, autism, and Alzheimer's disease.

## Conclusion

The results of the present study verify and expand upon the theoretical model of spatial ability proposed by Hegarty et al. (2006). Our analysis revealed a shared neural basis between large- and small-scale spatial abilities, as well as specific yet independent neural bases underlying each. Based on these findings, we proposed a more comprehensive version of the behavioral model (Figure 5). Our results further suggest that there is no absolute difference in cognitive strategies adopted during large- and small-scale spatial tasks.

## Ethics Statement

This study was approved by the Human Research Ethics Committee of Shaanxi Normal University.

## Author Contributions

YL, FK, MJ, YL, JL, and XY design of the study. YL, FK, MJ, and YL data collection, coding, and data analysis. YL, FK, MJ, and XY paper writing and revising.

### Conflict of Interest Statement

The authors declare that the research was conducted in the absence of any commercial or financial relationships that could be construed as a potential conflict of interest.

## Supplementary Material

Supplementary Table 1 PRISMA 2009 Checklist.

Supplementary Table 2 Samples of coordinate.
