# Supplementary material for: Shared and Distinct Neural Bases of Large- and Small-Scale Spatial Ability: A Coordinate-Based Activation Likelihood Estimation Meta-Analysis
Source: Front Neurosci. 2019 Jan 10;12:1021. doi: 10.3389/fnins.2018.01021 (PMC6335367; doi:10.3389/fnins.2018.01021)
Supplement: Supplementary Table 2 — Samples of coordinate. [file Table_2.docx]

**Large-scale spatial ability**

// Reference=MNI

// Nemmi, 2007

// Subjects=19

-14 -58 16

42 -80 34

-34 -86 28

-20 -82 -12

-24 -12 54

64 -50 14

-46 -60 20

52 34 -4

4 62 22

-50 32 -12

38 -8 -42

8 -50 34

-42 24 48

// Ino, 2002

// Subjects=16

-20 2 58

-40 -78 30

42 -74 32

-6 -52 8

18 -48 16

16 -52 16

-10 -62 20

10 -46 6

26 -26 -22

34 -68 -38

// Hartley, 2003

// Subjects=16

42 -12 12

30 -33 -3

-9 60 30

-6 39 48

-54 -21 -15

-36 -9 -39

-30 -18 -15

6 -66 33

-51 -48 30

48 -48 15

-42 21 12

-51 33 -9

-18 60 18

-27 36 -15

0 -51 39

36 -42 -15

0 -72 6

-63 -54 6

33 -12 -30

-33 -9 -30

51 -6 -12

45 -48 -24

21 24 6

// Baumann, 2010

// Subjects=17

-6 8 54

32 -60 56

-20 -78 18

40 -6 50

-44 -80 14

-48 -6 52

-44 -52 52

4 -64 4

-30 -4 58

34 -48 -40

-38 -44 -42

-32 -64 54

26 -80 22

-14 -80 54

6 -70 -24

-48 6 32

-10 -76 0

-40 -66 -30

38 -52 54

32 24 -2

-34 24 -2

-34 -54 48

-34 -50 -36

4 14 52

30 -6 54

-28 0 66

-28 -78 32

38 36 22

-12 -14 2

38 -44 -36

-12 -14 2

38 -44 -36

16 4 12

28 -42 -10

4 -52 -24

10 24 30

-14 -70 14

-14 -78 38

-48 4 30

12 -76 12

-12 -88 -8

-8 -74 -28

54 8 24

8 -70 -28

22 -54 56

-34 -2 56

-2 12 48

-8 2 58

10 -62 54

10 -66 60

-42 16 -6

-12 -68 64

32 -4 58

36 22 -6

4 10 56

22 -68 34

-38 -40 40

-16 6 8

-28 0 66

40 -44 -34

-34 -50 -36

-20 10 0

30 -58 -32

36 36 24

// Xu, 2010

// Subjects=20

29 55 -5

25 8 54

47 13 30

0 13 46

33 22 -6

-30 23 -2

14 -59 15

-21 -43 -14

25 -49 -10

33 -80 17

-32 -86 23

-24 -45 -15

26 -22 -11

7 -17 9

-7 -17 9

26 57 -8

28 38 31

28 55 21

26 7 53

28 38 31

48 12 29

5 32 31

32 22 -3

41 -79 18

-31 24 -2

12 -77 43

-3 -67 54

8 -49 -1

-21 -45 -14

26 -49 -10

-32 -86 24

-35 -76 -22

7 -19 9

30 57 -5

49 21 34

32 24 -6

-30 24 -7

0 12 47

12 -77 43

-3 -67 54

-9 -52 -1

34 -84 30

-31 -83 22

-32 -62 39

-24 -45 -15

-36 -74 -21

8 -13 7

30 -94 -12

-30 -94 -11

25 -97 -12

-21 -94 -10

34 -41 -2

-28 -41 1

13 51 30

-19 57 26

59 -57 -5

-39 18 40

48 46 -14

54 -41 39

-55 -52 36

31 -40 1

34 27 43

58 -42 19

5 48 25

-58 -44 34

0 -17 46

3 -54 63

2 -11 41

1 -38 29

-12 -38 38

-37 -36 57

62 -52 3

52 -61 0

-6 -50 52

-51 -63 7

2 -83 22

-7 -76 14

-17 -64 -4

25 -20 -18

-11 10 0

-4 -17 2

-36 -50 -26

// Schinazi, 2010

// Subjects=16

12 -31 1

-17 -33 4

11 -46 0

17 -53 12

-22 -50 6

40 -75 33

-46 -74 33

14 -57 26

-21 -63 26

3 -61 59

17 -61 63

-16 -60 63

-6 7 54

14 6 52

-27 1 58

2 -66 3

-21 -69 42

12 -69 51

9 -42 18

15 -54 18

-12 -54 18

27 -63 39

33 -75 27

-33 -78 24

-12 -51 6

-9 -69 -3

-27 -51 -6

27 -39 -9

-33 -42 -3

-21 -33 0

21 -30 3

6 -45 6

// Latini-Corazzini, 2010

// Subjects=16

3 -66 48

-3 -63 54

9 -51 9

-12 -54 18

27 -63 36

-15 -54 9

-9 -69 -3

-27 -51 -9

27 -39 -6

-36 -42 -3

3 -21 39

60 -36 24

6 -78 12

21 -75 -3

// Rauchs, 2008

// Subjects=16

10 -90 -4

20 -94 6

22 -92 20

-46 -76 2

26 2 56

-32 -64 -32

24 -28 -4

-28 -4 54

-18 -58 14

22 -58 16

-14 -18 44

-42 -28 44

0 -74 -32

8 -68 -42

-22 -28 -8

-18 -64 66

34 -46 50

-26 -48 -16

38 16 -28

4 -90 34

-34 52 -4

-10 22 46

8 26 40

-2 38 40

46 38 26

42 26 26

12 -82 -28

-38 28 22

-22 -48 -10

50 10 34

12 10 16

-60 -58 -14

-16 -76 2

54 -50 34

6 -10 -6

26 64 10

-6 -66 24

-4 -68 62

4 -66 62

20 -56 6

14 -60 -32

-10 6 16

20 -76 -2

28 -66 -8

66 -28 -18

20 -60 54

-60 -36 -12

-36 -74 48

-22 -48 -14

30 -50 -16

48 42 -16

-22 50 42

34 16 -26

-50 34 30

12 -26 2

44 28 20

-2 -66 56

24 16 64

54 -52 36

-6 46 32

-14 -58 12

50 -72 24

// Wolbers, 2007

// Subjects=13

-36 -76 -12

-32 -90 -4

-42 -66 6

-22 -66 34

-30 -62 52

-42 -36 50

4 24 50

36 -90 0

46 -54 -18

40 -60 -18

50 -58 6

26 -62 32

32 -56 48

46 -34 48

28 4 68

-4 24 40

// Wolbers, 2005

// Subjects=11

30 54 0

22 52 2

28 -2 44

42 8 32

-46 6 36

34 -50 46

54 -38 52

-32 -56 56

-30 -48 44

18 -68 60

-14 -54 58

28 -76 46

-16 -74 36

4 -62 24

-22 -12 -14

-20 -12 -18

24 -4 -18

// Rosenbaum, 2007

// Subjects=7

3 12 52

34 -5 38

28 -37 -13

32 -68 -19

-32 -60 -19

7 -56 15

-14 -57 11

33 -74 13

-31 -84 13

// Brown, 2010

// Subjects=20

-18 -36 -2

18 -34 -2

-28 -36 -22

24 -38 -18

-8 -4 -12

12 -2 -12

-12 -4 -2

14 -2 -2

26 -14 -4

-12 -20 -14

10 -20 -14

-4 -8 -52

10 -12 -50

-34 -48 -6

24 -56 -8

38 -22 -26

-30 -2 -54

36 -48 -26

26 -10 -54

-30 -24 -2

36 -26 -0

-46 -66 -10

44 -62 -14

-28 -44 -12

28 -60 -14

-26 -62 -54

-28 -76 -34

42 -70 -32

-6 -54 -54

12 -62 -56

-10 -46 -2

8 -48 -2

-12 -62 -2

6 -70 -2

-44 -76 -12

46 -76 -10

-4 -98 -2

10 -64 -18

-30 -56 -30

36 -52 -32

22 -32 -8

18 -40 -12

-8 -6 -16

14 -4 -14

-6 -6 -4

10 -8 -4

-20 -46 -16

22 -44 -14

-10 -30 -22

6 -20 -44

-44 -2 -54

-36 -30 -20

-34 -48 -8

42 -46 -22

12 -12 -58

-24 -4 -60

-32 -26 -0

34 -26 -2

-36 -66 -48

-34 -52 -44

-30 -82 -34

36 -72 -42

46 -42 -46

-4 -70 -44

8 -66 -46

-6 -70 -4

4 -72 -4

-8 -76 -32

10 -72 -30

// Iaria, 2007

// Subjects=9

-12 -60 6

-24 -22 -16

-26 6 52

-32 40 22

-34 -46 32

-8 -54 -18

-16 -58 20

-24 -38 -12

-26 -10 48

-6 8 56

-34 38 12

-30 28 32

-16 20 32

-32 -38 40

-16 -78 -8

-2 -14 -18

-4 -72 -18

20 -54 2

34 2 50

12 40 44

40 20 -10

56 -40 -2

54 8 -20

42 -48 30

46 -44 -28

2 -88 6

4 -72 -10

16 -54 14

24 -38 -6

24 -36 -2

34 30 30

28 30 2

36 -64 46

8 -18 20

6 -68 -22

// Grön, 2000

// Subjects=24

-6 -76 -2

-40 -74 -4

-16 -74 38

-20 -60 60

-16 -54 18

-22 -44 -4

-24 -4 5.81

40 -80 20

22 -74 42

18 -52 16

22 -44 -6

22 -36 -8

16 -66 0

30 -44 60

28 -32 -8

6 -24 -4

28 6 52

-18 90 30

-6 76 2

-10 74 52

-8 48 10

-4 24 -4

-12 86 34

-16 48 2

-18 32 -4

32 86 26

14 74 0

20 74 42

20 56 18

22 38 8

24 28 6

4 -24 -4

8 -60 66

-46 -68 4

-24 -46 -4

-10 -58 60

-18 -52 18

-4 -26 -4

-20 -60 64

-18 0 54

40 -76 36

14 -60 -4

26 -76 54

18 -48 14

22 -44 -8

26 -32 -6

10 -24 -6

24 -58 -12

30 18 56

48 32 26

44 44 22

// Weniger, 2010

// Subjects=19

20 -38 -7

-18 -43 -5

24 -49 -8

-24 -49 -5

9 -46 4

-15 -52 4

27 -73 34

-21 -73 25

9 -67 1

-6 -79 -3

39 -58 7

-39 -61 7

29 -76 22

-21 -79 21

-33 -31 49

-42 -25 43

-30 23 4

// Pine, 2002

// Subjects=20

0 -86 -32

22 -4 -12

-52 38 2

-48 -62 -34

-8 -68 -14

8 -6 4

-4 -42 42

-32 48 34

-18 -64 -26

24 0 24

22 12 28

-4 -42 0

-38 -4 -44

22 -2 8

36 1 52

26 -48 6

22 10 14

-40 -64 -14

-54 28 24

-10 32 40

-52 28 14

-20 -70 -14

-50 24 2

-38 -48 -32

-18 14 18

14 2 48

-10 18 44

// Shelton, 2002

// Subjects=12

-30 -54 -9

24 -69 0

-6 -81 0

6 -45 54

11 -60 30

-18 -63 60

15 -63 57

33 -75 36

-21 -30 48

33 -75 36

-12 15 54

30 12 51

30 48 12

33 45 36

15 39 24

-42 15 21

-18 -42 -9

21 -42 -6

-45 -9 9

-30 -66 -15

24 -72 0

-18 -81 -9

-6 -81 0

39 -81 21

33 -78 39

15 -93 24

-18 -66 60

15 -63 57

-15 15 57

-24 0 60

-51 21 33

30 12 51

24 -30 9

-36 -15 18

45 -15 15

-12 -45 60

9 -48 69

-12 -42 -18

21 -39 -12

21 -78 48

24 -60 21

-9 -18 48

18 -84 15

54 -51 15

54 -48 24

-33 -57 -15

-45 -60 0

39 -63 -3

-24 -66 51

36 -81 21

-33 -9 27

-6 39 57

// IglóiI, 2010

// Subjects=19

-21 -15 -15

30 -6 -15

18 30 9

18 27 0

12 9 -12

-18 3 -21

-3 42 0

-12 51 42

-12 42 36

-3 33 -12

42 -12 0

-42 -6 -3

0 42 -15

0 -54 30

-54 -3 -15

-63 -21 -12

-42 18 -33

-12 -48 39

27 -81 -36

-45 -72 -36

-33 -81 -33

-6 -81 -6

6 -81 -6

24 -24 -9

12 9 -12

-12 6 -12

18 27 0

18 27 9

3 33 -3

-24 60 3

-15 63 3

66 -6 -9

3 -63 36

-9 -57 45

6 -69 30

-9 -42 -12

-45 -72 -36

12 -90 30

12 -84 39

-6 -81 -6

-18 -69 -3

6 -78 -3

18 -69 -3

-21 -15 -15

0 27 -6

12 6 -15

21 -6 27

-3 60 18

39 -12 6

42 -21 -3

-42 -21 0

57 -3 9

57 -18 -6

39 18 -33

-48 -33 6

-66 -36 12

-42 18 -33

-33 24 -30

30 0 -21

30 -6 -15

21 -33 72

-42 -15 33

48 -15 45

42 -15 36

36 -18 45

9 -81 27

36 -75 -39

27 -78 -39

27 -87 -36

6 -84 -6

24 -39 -6

33 -45 -9

18 -54 15

-15 -57 18

-36 -57 42

-42 -48 42

-30 -69 36

48 -72 30

0 -75 45

-9 -69 48

-15 -84 30

36 -66 30

42 -66 24

3 15 51

39 60 0

-30 21 0

33 24 -3

-36 -42 -33

-27 -36 -39

15 -45 -45

12 -78 -21

39 -66 -27

39 -75 -24

-18 -75 -9

24 -24 -6

// Marsh, 2010

// Subjects=25

-44 -21 1

65 -26 16

-55 -27 11

63 -25 12

50 -36 48

40 -50 47

-34 -30 -15

38 -26 -19

-28 -22 -21

36 -24 -24

8 -69 20

6 -69 26

-18 -64 5

46 -12 37

6 -16 36

-14 21 -1

-16 14 10

-32 -16 -13

-8 14 9

-20 4 5

32 -14 -18

-22 4 2

28 -5 9

-4 16 -1

0 0 7

-20 -12 -16

24 -1 -13

// Rodriguez, 2010

// Subjects=11

-27 12 54

27 -57 -12

24 -63 54

-27 -63 51

-36 -84 27

-15 -66 18

27 -36 -3

-27 -75 -6

27 -84 -15

-33 -69 45

21 -3 -21

27 -9 -18

39 -87 0

-42 -81 -3

-30 -24 -21

42 3 39

-24 -54 9

21 -45 12

39 -45 60

-63 -24 30

66 -27 27

33 -75 24

-30 -45 42

-6 -45 27

// Orban, 2006

// Subjects=24

30 -30 0

-26 -30 -4

30 -46 -10

-28 -50 -8

30 -50 -16

-28 -54 -11

12 -96 -8

-4 -90 -6

22 -100 -2

-20 -90 8

24 -100 10

28 -90 30

-36 -90 18

-30 -92 24

22 -58 18

-22 -62 18

24 -76 42

-12 -84 48

22 -66 60

-14 -52 74

26 4 58

50 38 32

34 -38 -40

-34 -42 -40

// Moffat, 2006

// Subjects=30

36 -76 36

10 -74 44

-8 -76 40

-32 -90 22

-28 -80 24

-28 -74 32

-28 -44 -18

20 -32 -26

4 18 48

-6 12 50

-36 -64 40

14 -54 8

6 -44 16

26 24 50

-28 -2 60

28 -76 14

-32 -92 8

10 -40 0

-20 -36 -6

34 -84 -4

-10 -52 2

20 -32 -4

20 -32 10

-26 -84 -16

-22 -34 12

32 20 52

-44 16 50

28 10 58

-30 24 52

36 -72 32

-26 -72 32

26 -82 42

-2 -70 42

28 -88 28

-32 -86 24

32 54 6

-34 56 14

38 38 18

42 38 32

-38 12 36

-40 14 26

34 24 -8

26 -30 -32

-16 -66 14

14 -66 12

50 40 18

// Lambrey, 2010

// Subjects=18

21 -57 6

-51 9 0

12 -50 57

33 -51 63

15 -51 12

-45 -15 15

-18 -9 -15

30 -27 -18

-15 -54 6

-33 -3 9

-21 -30 -9

-27 -57 51

27 -54 9

-16 -54 70

38 -66 52

10 -46 26

-12 -52 24

-26 24 54

32 38 44

-48 34 14

// Whittingstall, 2014

// Subjects=18

-26 -43 -14

-9 -62 63

-38 -79 29

24 -39 -14

17 -54 16

40 -80 25

14 -48 -53

-11 -53 6

7 -60 61

-21 11 60

-15 -47 -52

30 -2 62

-30 34 40

-31 64 3

// Pintzka, 2016

// Subjects=53

15 -32 -13

18 -23 -18

18 -5 -12

22 -11 -15

28 -7 -10

30 -20 -14

-33 -37 -13

-26 -45 -6

-21 -38 -12

-10 -45 -5

-10 -11 -11

-15 -6 -14

-14 -14 -19

-15 -14 -19

-25 -20 -18

-28 -24 -14

-15 -6 -14

-19 -10 -12

18 -5 -12

23 -6 -10

22 -12 -16

23 -19 -18

23 -17 -16

// Jandl, 2015

// Subjects=26

3 -70 49

-3 -76 52

-48 -46 37

-24 2 58

-33 32 46

-48 23 37

30 5 55

33 38 46

36 35 37

-39 56 -5

-42 44 7

-45 50 -8

// Ledoux, 2013

// Subjects=22

10 -68 58

-14 -72 54

18 -76 54

-26 0 62

-38 -28 54

26 4 62

30 24 -2

6 20 46

6 32 34

-26 24 2

42 44 22

-50 -24 38

6 -72 -2

// Ganesh, 2015

// Subjects=23

-14 -48 62

66 -38 30

-46 -60 24

32 -32 -20

-4 -10 74

-32 -40 -12

-12 -80 42

-32 32 28

// Clemente, 2013

// Subjects=14

10 -91 26

27 -42 13

24 -98 0

-43 -49 -37

-8 -74 -25

-22 -84 21

3 -70 5

24 0 55

48 -70 -4

3 -74 0

// Mazzarella, 2013

// Subjects=20

48 -70 10

48 -73 -11

45 -64 -17

-51 -76 1

-18 -94 -11

-39 -79 -20

15 -67 52

27 -79 46

39 -46 43

-24 -1 49

-39 -1 49

24 2 55

36 2 52

-39 20 22

-54 23 28

-42 5 28

-51 -76 1

-21 -94 -11

-36 -49 46

48 -70 10

48 -67 -5

18 -97 -8

27 -1 55

39 2 49

-27 -1 52

15 -64 55

27 -79 46

18 -64 43

39 -46 43

-51 -76 1

-21 -94 -11

-36 -49 46

48 -70 10

48 -73 -11

18 -100 -8

15 -67 52

27 -79 46

39 -46 43

-27 -1 52

27 -1 58

39 2 49

-51 23 34

-39 17 22

-48 20 25

-60 8 22

-54 11 16

-27 -94 -11

-30 -97 1

-39 -85 -14

-6 11 46

24 -94 -11

39 -85 -17

3 2 61

0 11 61

-9 -1 64

12 -79 -14

-15 -88 -2

-15 -76 -8

24 -82 22

-21 -94 16

-18 -82 22

21 -94 -11

45 -76 -14

45 -67 -14

-24 -91 -14

-48 -79 1

-42 -52 -20

18 -61 55

30 -58 52

30 -52 46

45 -43 -23

24 -97 -11

51 -76 -5

-24 -91 -14

-45 -82 4

-39 -85 -11

18 -61 55

24 -97 -11

48 -73 -8

42 -79 -14

-24 -91 -14

-48 -79 1

-42 -52 -20

18 -61 55

30 -52 46

-24 -91 -17

-36 -67 -14

-39 -91 -8

36 -88 -2

36 -58 -20

30 -85 -17

-3 -37 -8

54 29 10

45 29 13

// Janzen, 2007

// Subjects=15

6.23 56.78 -10.31

12.54 -16.27 46.12

19.26 -9.4 -18.55

26.6 -69.68 36.59

28.2 -45.45 -2.81

24.62 -9.69 -21.98

36.11 -76.73 18.08

44.67 -20.46 22.45

60.47 -1.52 -5.47

-34.1 -82.4 18.72

-32.38 -40.76 -6.72

-28 -37.04 -1.57

-27.19 -36.44 62.23

-20.01 4.47 40.14

31 5.97 -27

23.67 26.31 -4.23

-40.26 -30.72 55.17

-1.23 18.16 -9.72

// Ohnishi, 2006

// Subjects=246

-27.27 -33.14 -28.86

-20.48 -36.26 -5.13

-13.75 -59.56 9.38

-15.27 -62.44 59

-7.19 -95.05 8.3

-13.1 -57.97 60.76

-37.13 -35.9 45.54

-46.54 -68 -21.72

-46.03 -83.82 15.7

-2.1 -36.09 -4.34

37.55 -41.63 -31.36

23.81 -38.33 -6.8

23.14 -57.12 21.96

19.33 -62.11 60.62

18.76 -101.34 8.48

34.42 -52.48 59.41

40.69 -42.01 44.82

35.62 -62.94 -18.02

55.27 -71.19 -4.09

14.22 -84.41 -5.44

4.31 -22.34 -6.93

56.43 21.37 21.47

// Janzen, 2010

// Subjects=20

21.47 -28.74 -18.92

22.43 -17.56 -25.64

23.61 -28.84 -20.06

33.42 -34.77 -15.16

31.36 -41.71 -8.84

33.42 -35.84 -15.06

25.98 -64.47 -10.98

// Iaria, 2008

// Subjects=10

67.35 8 27.09

70.62 -7.09 26.29

10.57 -30.13 58.73

49.74 36.16 7.8

12.63 -61.56 43.87

64.08 -52.38 12.95

53.82 -13.74 -20.95

37.62 -22.51 -22.05

23.74 -8.24 -6.42

-31.71 16.73 57.05

-7.67 -27.13 68.83

-47.51 10.52 3.03

-15.25 -38.21 65.57

-63.49 -56.75 7.71

-55.13 -44.66 -12.68

-13.32 -79.29 39.35

-37.65 -73.75 -3.37

-39.05 -20.34 -17.6

-25.24 -8.88 -33.53

9.29 15.39 52

59.82 -38.8 20.65

49.99 -58.27 8.17

12.16 25.78 25.15

28 -38.19 -16.97

37.24 -78.55 21.6

6.57 -12.89 2.18

18.28 -33.87 -16.11

-5.08 -82.28 7

-8.11 -88.38 22.23

-24.78 3.26 4.48

26.66 -53.24 -40.11

19.68 -51.58 -79.38

218.95 31.96 56.88

54.52 59.99 -36.1

27.31 7 -61.77

31.44 54.77 -67.69

84.13 40.01 -90.66

410.28 19.5 -109.87

119.52 -36.52 41.81

127.13 -1.55 -32.37

157.98 -25.8 -75.32

130.62 -19.78 -101.22

27.11 39.79 13.43

38.95 -27.23 -3.68

14.93 -29.18 -23.24

59.13 -64.72 -38.41

37.02 -48.87 -74.34

171.17 -55.77 -63.62

23.97 -11.28 -73.35

75.93 48.11 30.82

17.35 61 -69.19

135.19 41.2 -69.24

79.15 54.05 -54.99

48.71 -25.82 -.62

164.8 -48.03 30.98

55.37 -18.3 -69.83

64.94 -11.76 -80.73

25.79 -29.79 -18.89

20.81 -8.1 18.27

38.43 -67.21 -52.38

22.96 -14.06 -68.58

29.75 -33.2 36.29

17.15 18.32 -8.93

75.18 29.76 52.82

302.28 8.82 -22.93

314.09 20.8 58.6

37.38 46.1 57.45

28.92 60.16 -9.91

33.95 60.67 -39.18

139.39 -4.12 -3.19

101.35 -2.28 64.51

501.92 -33.35 31.63

43.03 -61.82 -29.46

195.29 26.84 -19.53

29.52 46.75 -50.05

177.24 -25.57 -5.07

105.3 -24.74 30.81

67.84 -21.11 60.22

178.9 -38.74 37.66

134.25 -53.61 7.4

56.36 -10.2 -75.13

32.17 -62.27 -33.71

148.12 -13.69 -83.07

195.29 26.84 -19.53

78.94 -9.45 .61

177.76 -32.68 33.72

// Rosenbaum, 2004

// Subjects=10

21.1 22.57 47.73

-37.07 12.66 59.79

28.05 -36.71 -12.64

-6.2 -49.75 10.53

43.87 -79.61 32.8

-31.68 -83.72 38.97

26.97 -36.71 -12.62

-19.01 -59.6 20.68

44.95 -79.61 32.78

26.97 -36.71 -12.62

-19.01 -58.54 20.58

44.93 -78.65 31.57

-33.83 -85.85 39.22

21.11 21.51 47.83

-36.09 9.71 51.1

28.05 -36.71 -12.64

-19.43 -44.12 -8.85

-19 -58.43 21.69

49.21 -70.23 29.54

-32.77 -84.89 37.99

24.85 -43 -10.84

-22.65 -45.09 -7.58

-10.49 -56.15 11.23

40.62 -84.08 31.06

-35.25 -96 11.12

-31.09 -93.94 -1.49

14.31 -95.8 -.95

37.7 -51.44 -21.43

24.85 -43 -10.84

-36.32 -93.77 12.03

-35.44 -87.57 -2.04

19.55 -46.65 -3.66

// Hirshhorn, 2011

// Subjects=13

-42.93 -76.13 5.92

-22.93 35.95 -13.36

-52.37 -6.62 -42.26

-27.46 28.23 53.6

6.28 72.5 -2.9

45.53 -7.88 7.74

-54.11 -19.88 -11.78

-0.01 18.13 1.46

-44.31 -1.79 -2.53

-48.99 20.68 -25.97

52.85 33 -3.15

56.27 -33.45 -2.24

59.67 -53.02 6.37

-58.84 -59.77 32.59

18.06 -91.95 40.06

50.49 -3.09 58.73

-10.2 -48.44 36.24

-25.53 42.95 38.66

34.5 -23.82 -12.9

45.25 -38.44 -20.6

-34.78 -15.22 -20.42

-57.44 .21 -14.83

-60.6 30.37 -2.08

-53.99 -17.88 -1.9

-20.87 29.09 62.37

-8.67 -31.93 74.92

66.47 -20 37.72

33.7 -74.25 -1.17

-20.77 35.96 -13.39

67.03 -14.06 -2.1

-1.09 20.26 1.27

26.06 -42.06 -.87

50.39 16.57 -29.5

// Grön, 2000

// Subjects=24

-19.85 -89.51 33.74

-5.16 -79.6 1.14

-41.93 -77.81 -.65

-15.42 -73.36 45.52

-19.49 -56.2 68.54

-15.78 -54.17 21.21

-22.6 -45.84 -4.15

-24.79 -2.26 2.78

44.86 -81.4 25.12

25.69 -72.8 49.24

20.92 -52.12 18.14

24.9 -45.88 -7.19

24.84 -37.58 -10.26

18.6 -68.68 1.89

34.46 -38.99 65.92

31.31 -33.3 -10.79

7.56 -24.46 -6.78

31.99 13.35 51.82

-18.36 100.23 19.43

-5.73 82.47 -10.41

-9.35 85.52 45.38

-7.66 53.52 1.45

-3.44 26.55 -11.64

-11.81 96.41 24.18

-16.42 52.66 -7.28

-18.59 35.01 -12.22

35.62 95.75 14.47

15.86 80.22 -12.79

22.93 84.6 33.72

22.67 62.96 8.97

24.76 42.79 -.28

26.94 31.95 -1.48

5.4 -24.47 -6.74

10.84 -55.47 74.68

-48.32 -70.62 7.71

-24.76 -47.97 -3.9

-8.69 -54.04 68.14

-17.94 -52.05 21.04

-3.23 -26.63 -6.38

-19.43 -55.79 72.98

-17.66 7 55.53

45.06 -75.48 42.46

16.35 -62.72 -3.14

30.19 -73.67 62.7

20.88 -48.07 15.5

24.88 -46.09 -9.41

29.18 -33.1 -8.53

11.86 -24.65 -9.07

27.04 -61.38 -12.42

34.15 26.53 54.96

53.13 38.38 19.85

48.7 50.71 14.22

// Iaria, 2003

// Subjects=14

21.5 -60.93 61.59

33.7 40.64 22.19

27.77 -5.61 56.01

6.01 10.71 48.04

10.36 2.22 48.81

14.48 24.8 37.54

14.54 9.91 39.01

31.33 6.23 -1.25

18.66 -4.7 20.21

33.01 -38.95 -48.36

-28.19 -56.65 64.25

-33.25 31.9 24.2

-28.46 -1.76 54.34

-32.57 -17.75 67.2

-4.65 -3.59 56.36

-32.97 -84.21 22.24

-31.39 5.6 -4.61

// Maguire, 1997

// Subjects=11

18.48 -38.9 -1.05

-29.12 -43.94 -6.47

20.56 -39.51 -7.75

-13.48 -59.71 30.69

-19.5 -67.25 65.15

8.65 -62.28 70.9

-8.94 -64.62 46.78

-42.61 17.75 -34.75

44.97 -80.57 34

-32.7 -82.14 44.44

-50.83 -84.18 -22.29

-29.12 -43.94 -6.47

9.85 -43.18 -.48

-22.72 -44.54 -13.24

16.65 -51.72 22.66

-0.05 -60.59 66.4

-34.1 21.2 -44.2

-42.36 -32.89 -25.26

-52.75 22.7 18.72

-25.26 31.4 -27.43

-17.74 19.55 52.05

-30.57 -88.93 40.59

-14.14 -44.93 -17.83

37.53 -83.4 -41.8

-56.8 -62.82 22.77

-38.39 21.4 -41.91

-48.53 -45.93 -3.7

-59.34 28.43 11.54

-7.81 -94.19 -39.96

39.56 -86.56 -52.72

-56.8 -62.82 22.77

-36.18 23.95 -37.72

-53.34 -1.43 -32.67

-11.08 18.69 65.47

37.39 -84.44 -52.9

// Kaiser, 2008

// Subjects=24

36.39 -75.72 40.39

-29.54 -70.74 39.9

11.75 -57.88 60.33

40.67 7.1 53.41

46.62 19.54 13.98

50.01 8.69 23.96

4.74 29.02 37.28

28.82 -34.63 -36.39

-12.25 -54.65 -41.55

-34.75 22.19 -10.67

-20.94 9.97 53.05

4.63 -23.25 17.81

10.88 -6.39 2.58

-43.98 8.36 25.59

// Hirshhorn, 2012

// Subjects=16

27.54 32.06 45.56

-24.3 34.21 48.47

-1.26 52.92 -5.32

-2.85 -58.79 16.97

42.9 -73.36 42.28

-39.25 -78.32 39.69

29.03 -25.42 -18.25

-27.17 -34.54 -20.88

-11.63 -97.06 -1.51

7.22 -53.31 -39.77

63.56 -5.95 -18.53

-63.89 -14.91 -15.47

// Powell, 2012

// Subjects=82

-11.56 -92.18 4.73

-4.69 4.92 55.52

-39.32 -0.08 49.88

-33.52 21.1 .62

-43.6 -34.76 46.66

16.48 -88.04 1.6

55.42 10.94 24.76

34.16 -71.89 35.56

36.51 -46.41 55.41

10.32 14.98 47.54

49.37 -34.01 49.48

34.17 .38 50.82

-17.76 -98.84 25.67

-26.85 -80.3 -5.15

-52.42 5.42 40.59

-28.26 -52.81 59.39

-4.74 11.09 52.67

-43.62 -37.09 44.65

-35.7 18.76 -1.35

-42.33 -69.88 -30.57

-29.41 -59.2 -31.85

16.57 -96.13 6.88

51.04 10.51 20.4

51.24 5.37 34.35

36.51 -46.41 55.41

6.14 7.29 57.34

35.58 23.27 -3.01

14.5 27.14 39.55

// Etchamendy, 2012

// Subjects=30

28.98 -7.95 -35.78

-31.26 -58.8 -16.05

-35.25 -86.78 29.37

-38.01 57.89 4

-19.56 28.69 46.58

51 31.78 29.84

36.15 -31.04 13.11

12.28 1.86 12.73

47.25 11.99 36.45

35.82 15.14 47.88

36.97 -18.7 -21.41

-20.44 67.75 -6.35

-7.71 22.19 -8.67

-5.16 -79.6 1.14

-41.93 -77.81 -.65

-15.42 -73.36 45.52

-19.49 -56.2 68.54

-15.78 -54.17 21.21

-22.6 -45.84 -4.15

-24.79 -2.28 2.57

44.86 -81.4 25.12

25.69 -72.8 49.24

20.92 -52.12 18.14

24.9 -45.88 -7.19

24.84 -37.58 -10.26

18.6 -68.68 1.89

34.46 -38.99 65.92

31.31 -33.3 -10.79

7.56 -24.46 -6.78

31.99 13.35 51.82

// Lee, 2005

// Subjects=10

10.03 35.86 29.79

-48.26 11.95 29.79

50.42 53.98 -19.75

56.24 19.92 5.93

52.97 39.36 7.43

-50.52 17.7 22.54

-5 32.07 36.03

63.39 -15.57 50.78

58.9 -14.6 38.44

21.57 -61.48 67.24

38.65 -53.09 52.68

-10.89 -64.13 63.58

-23.88 -46.85 65.45

-19.65 -63.93 54.74

42.93 -38.08 52.24

-32.73 -40.79 50.43

-41.44 -33.68 46.52

38.05 -92.63 -2.79

46.59 -82.38 -8.43

-29 -93.39 -7.18

-37.5 -93.44 4.18

-26.69 -99.89 3.51

42.52 -88.18 10.14

46.95 -82.97 19.63

40.75 -73.27 43.43

50.88 -73.86 -9.35

42.73 -81.2 27.37

-52.68 -75.31 3.76

-50.42 -80 10.91

-44.1 -82.28 -2.42

-28.49 -72.01 37.76

-37.2 -83.5 30.08

60.75 -66.19 3.17

56.23 -66.6 -12.4

-55.91 -68.63 6.52

-50.82 -61.11 -16.72

37.55 -41.63 -31.36

18.3 -47.9 -16.97

-11.83 -89.8 -15.67

-20.5 -98.75 -19.12

// Lux, 2003

// Subjects=14

-46.32 7 11.2

-6.78 3.2 60.21

33.53 24.09 5.91

51.03 -68.35 3.55

34.32 -46.62 53.23

-37.08 -43.24 47.39

-39.73 -79.73 1.74

-31.42 -66.88 -22.09

-31.8 -74.04 -52.75

39.74 -61.08 -32.84

// Kesler, 2004

// Subjects=13

-46.12 -81.25 8.72

25.85 -64.01 62.94

40.68 -39.88 44.61

48.72 -73.86 -9.31

-41.74 -81.89 13.19

34.46 -68.54 59.88

6.07 14.42 53.27

-24.27 3.81 44.76

**Small-scale spatial ability**

// Reference=MNI

// Gogos, 2010

// Subjects=10

36 -73 15

26 -69 48

-34 -81 19

14 29 36

-18 2 50

-37 -46 -30

-20 -20 3

-36 -48 13

28 -3 55

36 -58 -24

59 -29 44

26 -53 65

53 -44 48

-32 -51 62

-44 -48 56

8 27 43

// Weiss, 2009

// Subjects=16

-27 -3 51

33 3 45

-48 0 54

42 6 60

24 3 69

-54 6 45

-60 9 30

57 12 39

-3 15 48

9 12 48

15 24 39

-33 27 -3

30 30 -6

45 15 27

42 12 36

30 51 39

-21 -63 54

24 -60 51

-39 -39 45

30 -51 45

-42 -60 -18

48 -81 -12

27 -69 36

-45 -57 -36

-24 -33 -9

-39 -21 -12

-36 -30 -6

33 -24 -12

27 -6 -18

-27 -57 12

-12 -60 33

-3 -27 42

-48 -60 27

-39 -12 -6

-42 -3 -12

27 -3 48

-45 9 27

-57 12 30

45 12 21

12 3 60

-6 24 45

9 21 45

-30 24 -3

-42 18 -15

51 42 18

-36 33 -3

39 27 15

-15 -75 51

15 -75 54

24 -66 45

24 -60 39

33 -54 54

33 -39 42

-36 -39 48

-45 -69 -6

-45 -51 -12

48 -60 -9

33 -72 18

// Wraga, 2010

// Subjects=18

42 -57 -35

59 -20 12

30 -81 7

-39 -45 38

12 22 32

-39 43 -5

45 -54 44

-36 -76 -1

-41 14 -8

41 -6 3

-33 13 19

-21 -45 -31

-39 8 38

-39 44 14

-27 -80 29

-62 -48 30

-45 0 3

15 -37 -33

-36 -23 65

39 -23 65

65 -42 33

-15 -74 -22

-30 -71 -22

-3 -64 53

-56 -36 27

0 52 -8

12 -100 -10

62 -9 -15

12 50 17

-3 -29 62

21 22 38

3 -60 17

15 -45 33

-6 62 19

21 34 39

24 37 48

48 -71 28

6 -22 26

-12 -34 58

4 -18 64

33 36 20

9 -99 0

18 -29 37

-30 -53 -10

-12 -34 58

-6 -20 67

56 5 -18

-33 13 -31

12 -100 -1

33 45 36

// Johnston, 2004

// Subjects=9

6 -62 58

2 14 54

-42 18 2

// Jordan, 2001

// Subjects=9

-28 -64 48

28 -64 48

32 -80 16

36 -80 0

48 8 24

40 -64 -28

-28 -64 48

24 -68 48

32 -80 16

44 -64 -16

52 8 28

-32 -4 44

44 -4 56

-28 -64 48

24 -68 48

32 -80 16

44 -64 -16

48 8 24

32 -64 48

-48 -32 48

-40 -12 56

36 -60 -32

28 -68 48

48 8 24

32 -76 -24

32 -80 16

-28 -64 48

36 -52 52

-24 -64 48

24 -56 56

32 -48 44

-32 -4 40

-24 -64 48

24 -56 56

-28 -64 48

24 -64 48

// Seurinck, 2005

// Subjects=24

26 -1 52

-20 -3 57

-48 7 29

51 5 20

22 -53 58

36 -35 40

-20 -48 54

-38 -35 39

42 -64 7

-44 -70 7

42 -72 7

34 -82 -3

-42 -72 -3

-34 -82 -3

-24 -3 55

28 -1 57

-24 -57 58

-46 -33 42

24 -55 60

36 -40 50

-40 -70 -2

-28 -80 -13

28 -76 -11

38 -80 -8

24 -1 53

-20 -3 48

10 -55 62

30 -44 54

-26 -53 60

-28 -48 54

-24 -3 57

28 1 53

-12 -61 58

-48 -29 46

14 -60 58

36 -39 41

// Creem-Regehr, 2007

// Subjects=16

-38 -88 -4

30 -88 -6

-30 -6 62

-4 -80 -20

12 -80 -24

4 -74 -16

-38 -40 40

-34 30 0

-26 32 6

-54 2 30

-4 10 48

-20 -72 50

30 -32 -2

28 -64 52

32 -68 26

-40 -88 -4

30 -88 -10

8 -70 -24

-6 -24 -8

32 -54 56

28 -64 44

28 -34 0

-26 -32 -6

-36 -30 34

34 -74 26

-34 -78 14

-14 -62 54

38 -74 16

52 -72 16

-18 -76 30

-42 -82 16

-22 42 4

-28 -36 30

12 -48 50

16 -68 60

// Sluming, 2007

// Subjects=10

-39 91 2

-18 -79 59

-21 82 32

-6 70 62

42 -49 59

33 -61 50

27 -61 56

36 -88 32

42 -85 2

-45 -43 53

-61 -70 -1

-48 5 38

30 -1 65

0 17 53

// Wolbers, 2003

// Subjects=13

21 -42 63

33 -33 54

33 -39 63

-9 -36 60

-27 -39 66

-21 -54 69

42 -15 63

3 -6 51

-24 -51 -27

-36 -21 63

-6 -3 51

24 -48 -27

// Keehner, 2006

// Subjects=14

42 -44 57

30 -59 44

-42 -45 38

56 -21 45

-48 -33 49

-30 -58 61

53 -41 52

48 39 20

27 -73 56

50 13 32

// Lamm, 2007

// Subjects=13

-26 -92 22

12 -68 8

24 -68 32

-40 -68 -8

24 -56 -10

52 -68 10

26 52 -22

-16 16 -8

-14 -84 46

32 32 -6

-12 -72 8

-42 -22 42

-34 10 -2

34 4 0

48 -4 8

-24 22 -26

// Vingerhoets, 2002

// Subjects=13

17 -89 -19

-25 -48 31

29 -50 50

-24 -9 50

26 -9 52

33 -85 -12

-4 -31 -25

1 -40 -27

-24 -69 -23

24 -60 -32

-29 -58 53

17 -67 48

-25 -3 51

-22 -79 22

24 -81 25

59 -36 -9

6 43 44

-4 -69 30

-3 -36 33

-1 -27 27

48 -63 16

-50 -3 -7

-8 57 15

4 37 49

-47 -60 34

10 -87 -5

45 -63 9

-40 -69 1

17 -62 2

-29 -54 -15

-29 -62 53

// Wraga, 2005

// Subjects=11

-12 -60 51

-4 -59 55

24 -63 51

-36 2 48

-55 -47 -14

8 -69 11

-4 -93 1

-8 -25 49

-40 -19 5

-48 -7 11

-20 -92 27

-20 -97 12

12 56 34

-16 45 38

-20 37 39

4 34 -22

-8 38 -12

55 -61 29

60 -53 25

24 -94 -5

-32 10 44

-48 13 29

24 -59 58

4 32 28

-4 29 35

-8 36 17

32 23 -15

-52 -51 -11

20 62 -6

40 -32 -22

-52 -32 -19

-28 -93 -2

-12 -97 12

52 4 0

-55 -13 45

-8 -9 59

-16 -13 -16

-32 -21 -26

-4 50 3

-55 -30 16

-44 -31 9

36 -21 42

44 -17 52

36 -17 49

55 -65 29

-55 -61 25

-59 -50 10

24 -17 -26

59 -9 -16

-59 -4 4

-55 0 -7

// Corradi-Dell'Acqua, 2009

// Subjects=17

54 -68 2

-50 -76 4

-4 -102 -4

-14 -92 -14

2 20 46

// Halari, 2006

// Subjects=19

44 -20 -6

0 -38 42

50 -10 2

-22 26 38

-36 12 42

4 12 48

54 -60 28

52 -58 38

60 -50 32

-40 -44 46

-30 -4 56

-34 -76 22

-32 -82 2

34 -70 22

-36 -4 52

32 -62 52

26 -54 62

16 -12 60

-20 34 40

-12 -44 44

40 -56 50

-28 -78 12

-38 -78 10

-34 -78 -2

-38 -40 56

12 -52 62

16 -58 58

// Creem, 2001

// Subjects=12

-20 -74 50

17 -64 61

-18 56 66

-43 -3 38

-5 -53 65

-25 55 27

-39 -47 -24

-25 -13 67

5 -69 -21

-8 -64 43

30 42 -14

-3 -93 26

-54 7 33

-33 10 29

-44 54 1

// Thomsen, 2000

// Subjects=11

22 -77 38

40 24 22

-20 -58 46

-38 20 22

// Lange, 2005

// Subjects=6

-46 -68 -6

34 -74 30

-42 -36 44

36 -40 38

-30 -50 54

24 -60 54

-22 -12 48

28 -8 48

0 14 50

-28 22 0

26 -66 40

-26 -60 58

26 -56 56

46 4 26

-24 2 66

6 20 44

34 44 38

// Logie, 2011

// Subjects=21

-26 0 62

26 0 64

-40 10 -4

42 -18 20

4 -86 12

-10 -64 -4

56 -28 30

60 0 -8

60 -46 -16

26 14 -18

0 -4 66

6 -18 34

18 -42 -6

-52 -50 0

-2 -22 -16

36 -54 22

// Levin, 2005

// Subjects=12

-30 -92 -6

-12 -100 9

40 -76 4

-30 -68 -7

2 -27 49

-38 -32 -22

-12 59 10

2 45 3

55 -14 -8

57 -43 28

34 21 -9

-14 -63 60

6 -64 29

30 12 42

38 -38 55

51 20 41

50 13 36

44 -78 -1

// De Lange, 2006

// Subjects=17

-18 -66 48

20 -66 52

-24 -8 54

30 -8 56

12 -32 74

34 -20 14

-14 -38 -32

// Ferri, 2014

// Subjects=18

48 3 57

-3 3 57

9 3 51

-54 -3 48

60 9 21

51 -3 36

-9 -36 48

12 9 36

-27 -33 48

30 -12 -18

48 -72 3

42 -54 -15

27 -78 -15

-12 -75 12

15 -72 15

-27 -93 12

-21 -57 -9

24 -51 -6

-36 -66 -21

27 -48 -30

-27 24 -3

42 21 -3

-3 12 48

-9 -93 -6

-42 0 33

// Kawamichi, 2007

// Subjects=14

10 -86 46

-14 -84 44

28 -4 58

-24 -8 50

-42 -74 -32

50 42 24

42 -62 -34

56 10 36

-48 10 24

8 12 48

-50 -48 42

32 -58 34

-30 -66 54

44 -62 -32

-24 -92 20

58 -22 36

-42 -74 -32

34 8 60

42 -38 30

44 -46 62

-30 -70 56

8 -80 52

44 -62 -30

-38 56 22

-42 -74 -32

34 62 14

54 10 34

-44 6 24

32 -68 -32

-24 -8 62

10 -86 46

40 -62 -34

-32 -68 58

-28 -86 12

34 -56 30

42 -42 36

-42 -74 -32

28 -64 36

38 58 10

-24 -10 54

// Jordan, 2002

// Subjects=24

32 -84 8

36 -76 -24

24 -64 52

24 -72 48

44 -64 -16

16 -84 40

-32 -72 -32

4 24 36

0 12 48

52 8 28

8 -20 8

-12 -24 8

-28 -88 12

-24 -72 48

32 -4 60

-36 -48 60

-56 4 28

20 -68 52

52 -60 -16

32 -88 0

-16 -76 52

-40 -48 44

-24 -76 32

28 -4 56

-48 -68 -12

-36 -76 -28

-28 -4 64

-4 16 48

4 12 52

52 8 24

12 -88 40

-36 -60 56

-28 -72 48

24 -8 56

-28 -20 56

0 12 48

// Lamm, 2001

// Subjects=13

-26 -82 2

38 -78 6

-24 -58 52

22 -64 54

10 8 52

-28 -10 64

30 -6 56

-48 4 28

56 4 28

36 14 2

-32 18 2

// Paschke, 2012

// Subjects=10

-12 -78 3

-9 -72 9

6 -69 0

// Milivojevic, 2008

// Subjects=14

15 -69 55

33 -51 55

27 -57 55

48 -63 -5

39 -81 10

30 24 5

54 9 25

27 0 55

51 30 25

3 15 50

27 -54 65

15 -66 55

3 15 50

27 0 65

33 18 0

// Stoodley, 2012

// Subjects=9

-4 -74 -32

32 -70 20

-34 -62 -6

34 -82 -4

40 -58 -8

26 -64 58

36 -40 40

-22 2 26

50 -46 -6

40 -40 32

-24 -62 52

-36 -84 0

-14 -62 54

22 -92 10

12 -66 48

12 -90 2

30 10 54

-34 -46 42

// Wilson, 2006

// Subjects=7

26 -86 -25

38 -52 55

19 -4 55

30 -94 10

19 -94 -10

-38 -64 55

-30 -90 15

34 -90 15

-38 -60 -30

26 -75 50

45 -68 -25

// Papeo, 2012

// Subjects=18

-30 -4 62

-22 -4 50

54 8 28

44 4 32

-42 36 34

-40 28 26

30 20 4

-34 16 4

-18 -24 10

18 -24 10

-24 10 4

-32 -92 6

18 -98 14

-24 -64 -52

12 -70 -50

// Schendan, 2007

// Subjects=16

-30 24 0

36 33 3

-45 6 27

-48 33 27

-27 3 63

24 9 57

9 36 42

-6 18 24

9 6 30

-24 -66 60

33 -75 30

-36 -78 18

-39 -54 -3

-33 -54 -15

-27 -48 -6

30 -42 -9

-42 -33 3

-54 9 -18

24 -21 6

-33 -30 3

18 0 3

3 -24 -12

0 -33 -33

6 -81 -33

30 -72 -33

6 -63 -39

// Seurinck, 2011

// Subjects=16

-40 -78 0

-48 -72 -6

-26 -94 12

-24 -78 36

-20 -76 46

24 -72 48

-16 -64 52

16 -66 50

-30 -54 62

28 -56 54

-40 -38 46

34 -40 46

-24 -4 58

30 -6 56

40 6 34

-10 20 42

// Seurinck, 2004

// Subjects=22

20 -3 63

-28 -5 57

26 -68 46

28 -50 54

-36 -52 62

-48 -74 4

-26 -72 37

4 -83 1

28 1 55

-20 -3 57

-48 7 27

20 -55 62

-20 -50 54

-38 -35 39

34 -83 1

-42 -68 -8

-26 -71 39

-12 -90 -6

-30 3 59

34 18 -4

30 -51 58

-20 -57 56

-48 -39 41

-30 -70 33

28 -66 38

28 -64 -2

34 -70 -5

38 -72 -8

30 -83 6

34 -74 28

-44 -76 -1

-40 -79 4

26 1 55

-20 -3 55

-50 9 29

-16 -43 70

24 -55 60

40 -40 48

-20 -57 62

-44 -31 38

-24 -66 38

26 -68 40

38 -72 -10

36 -74 -5

28 -74 28

-38 -78 -10

-36 -76 2

// Vanrie, 2002

// Subjects=6

27 1 54

-51 39 27

25 -57 57

37 -67 -24

13 -60 4

39 -36 39

34 -78 16

3 30 45

42 -66 -22

14 -64 48

6 -88 -4

-2 -40 70

-25 2 56

6 34 46

-52 24 42

48 58 6

23 -56 65

48 -26 54

48 -76 -6

48 -76 -6

14 -68 16

-54 -60 -2

28 8 58

48 44 34

32 -70 28

2 -98 -6

49 -26 41

34 -62 -22

-26 -60 60

56 22 26

32 -7 53

12 -74 59

-2 -90 24

9 -73 3

14 -72 -20

-32 -48 -18

-12 -50 -4

29 2 66

-56 -60 -2

4 28 50

54 26 44

56 10 32

35 63 -4

-44 -84 4

25 -62 62

30 -68 34

35 -79 -24

20 -82 -26

28 -5 60

60 14 26

52 -30 43

-2 6 58

0 30 46

16 -77 53

4 82 -1

8 -91 34

-32 -90 12

-30 10 68

43 -42 55

44 -70 -16

// Baumann, 2012

// Subjects=14

-32 -58 40

2 -68 38

-54 -40 -6

40 -52 36

// Bodin, 2010

// Subjects=11

-30 -90 16

-34 -88 -14

-36 -90 -2

-38 -40 46

36 -46 48

-20 -72 60

20 -68 58

-30 -6 -56

26 0 62

-58 10 34

50 10 26

0 10 50

-44 32 38

42 48 26

30 24 -8

-16 -14 -10

2 -78 -32

36 -54 -28

-24 -40 -46

20 -36 -50

// Gao, 2017

// Subjects=30

3 51 27

54 -21 12

-45 33 12

36 -54 48

18 -99 12

6 -51 27

0 9 51

6 -75 15

-3 48 0

-42 -15 45

// Goh, 2013

// Subjects=97

-33 -48 51

30 -42 45

-36 -93 9

36 -90 18

36 51 18

-36 -72 3

45 -72 -9

// Wolbers, 2006

// Subjects=16

-36 -40 46

-38 -42 48

32 -36 40

-42 -56 30

50 -58 36

46 -56 32

// Blacker, 2016

// Subjects=32

-29.8 -35.35 -26.65

-20.29 -42.52 -10.71

-20.71 -93.79 -11.62

-60.07 -7.32 65.33

-81.96 19.1 -1.04

-87.97 -29.44 8.38

-78.79 19.06 38.54

-41.6 -4.14 -46.93

-14.5 31.84 35.93

-17.57 32.51 66.68

-15.99 16.65 43.13

-18.41 -29.52 50.95

25.64 -82.86 -7.15

54.41 -7.99 70.37

84.84 -12.75 11.52

47.39 -39.11 74.53

16.97 -12.62 56.1

15.03 29.21 41.68

67.64 40.34 -14.32

24.46 -77.17 -14.2

6.72 12.69 -10.32

26.53 -95.84 27.79

20.42 -76.21 43.86

19.72 -98.3 9

35.5 -5.26 -42.82

-20.47 -89.96 -9.25

-9.08 3.35 -12.21

-16.55 39.15 55.39

-87.36 -30.04 5.66

-54.53 -73.52 45.39

-77.26 31.85 32.84

-89.95 25.1 10.67

-73.84 61.65 11.37

50.71 42.62 59.09

-19.31 -74.72 44.32

-39.57 -4.69 -46.33

-66.62 -49.92 45.7

// Newman, 2016

// Subjects=36

20 -30 -20

0 -38 -8

-36 -54 -10

-36 -46 -12

36 -42 -8

-8 50 32

-44 -6 24

// Hugdahl, 2006

// Subjects=11

25.63 -72.15 44.7

-19.64 -47.46 58.71

40.29 13.01 24.82

-43.09 24.44 13.9

// Schöning, 2007

// Subjects=34

-41.09 -49.69 70.51

-37.01 -47.08 52.25

-34.92 -36.64 48.94

41.03 -60.24 67.91

58.2 -39.12 63.29

12.97 -75.44 67.65

5.98 25.81 48.79

-21.88 3.35 62.69

-19.62 -6.86 68.14

-52.63 14.81 26.22

-37.61 18.5 18.88

-56.81 15.83 37.4

-27.22 30.72 -11.64

-31.39 27.49 -0.05

-48.34 36.51 28.48

-37.64 35.72 19.41

37.59 26.49 -14.57

44.39 39.85 13.13

57.57 37.09 28.86

34.33 3.76 63.93

50.73 -55.33 -17.9

52.9 -66.17 -19.11

-40.07 -66.91 -21.94

-46.44 -81.41 -15.91

-33.4 -69.9 -8.31

33.77 -96.69 -0.08

-5.56 -86.97 -31.74

33.26 -74.28 -35.9

39.66 -52.99 -38.12

33.15 -46.63 -38.64

-43.42 -50.94 57.22

-36.88 -59.22 60.17

-47.76 -40.11 58.47

-45.87 -26.88 39.2

25.87 -73.68 62.77

12.87 -71.81 60.57

34.14 -58.92 36.52

64.29 -24.57 34.85

-30.63 .36 54.17

-10.98 -.24 69.58

-21.8 -2.61 67.76

-25.22 27.36 -24.79

-50.55 14.2 19.52

-61.23 17.32 30.6

-31.34 30.03 4.18

61.78 14.38 15.35

23.47 9.89 61.27

36.52 3.97 66.12

46.85 7.07 29.77

-37.96 -60.73 -24.82

57.13 -58.06 -24.47

46.9 -51.61 22.13

-41.97 -89.07 -6.26

-40.2 -76.67 -34.41

-46.74 -61.8 -35.77

40.35 -91.58 8.27

48.77 -86.63 -8.05

33.77 -81.39 2.89

-18.38 -7.12 -3.57

-18.15 -29.48 9.85

-9.95 -83.15 -36.53

26.77 -74.3 -35.79

33.15 -68.52 -43.19

35.25 -53.63 -44.7

-28.49 -80.83 35.27

-26.29 -69.56 40.84

-28.54 -46.17 38.57

32.02 -60.85 38.99

38.54 -49.56 44.48

32.19 -70.44 51.14

-6.81 9.58 59.58

-48.43 16.12 17.06

-41.57 8 44.64

27.73 2.9 55.17

-29.47 23.5 -19.86

-31.66 32 -20.66

44.23 23.3 -3.16

37.65 33.49 -8.54

55.7 13.01 46.96

36.29 4.44 48.14

61.8 14.59 17.57

12.26 33.1 34.51

8.07 37.31 45.37

-6.81 9.58 59.58

-37.6 -93.1 -3.7

-39.63 -92.07 7.44

-33.46 -92.21 -17.3

35.34 -61.72 -39.42

48.69 -71.94 -11.74

46.26 -62.98 -30.52

-18.29 0.09 4.68

-31.68 -66.62 -42.28

-44.51 -72.22 -32.54

-44.63 -64.34 -40.04

7.3 -74.58 -37.67

16 -74.13 -33.38

// Suchan, 2002

// Subjects=10

38.67 63.69 -7.06

50.87 44.23 13.7

30.85 17.29 48.09

-32.83 10.99 53.16

11.93 2.12 1.72

45.08 -22.87 54.06

-34.92 -32.29 49.63

-28.21 -46.97 64.41

41.86 -51.16 50.19

32.23 -54.83 57.44

-22.85 -58.21 58.71

8.48 -59.16 58.27

-16.44 -62 52.25

-18.64 -74.33 46.79

33.89 34.44 36.25

-24.28 32.19 49.79

31.88 30.05 46.81

2.75 30.26 50.64

41.78 -30.95 48.19

8.44 -47.46 57.12

41.85 -50.1 50.08

-4.59 -54.51 51.31

34.01 25.49 43.86

-29.69 15.7 45.91

-41.63 48.08 48.51

-20.68 -61.39 58.99

28.96 -45.27 56.55

// Suchan, 2006

// Subjects=11

58.17 38.99 -8.31

13.98 33.07 -.25

10.8 28.06 3.66

46.8 5.59 25.44

64.98 4.3 10.69

55.19 3.75 5.31

65.17 25.42 29.89

53.44 41.83 45.27

50.18 47.13 44.8

55.48 56.21 38.21

30.85 60.01 57.31

17.91 66.66 60.23

36.88 55.54 20.67

43.44 46.31 24.83

14.76 35.6 61.12

40.65 23.36 55.16

17.01 92.75 -5.09

10.44 95.4 -10.84

3.97 100.9 -9.03

// Podzebenko, 2002

// Subjects=10

-30.54 -82.13 44.4

25.88 -75.81 62.99

-43.48 -51.36 52.78

39.77 -76.18 -33.58

38.75 -52.36 60.45

-37.43 -93.99 9.83

36.44 -5.37 58.08

48.64 15.22 2.04

-2.13 -59.98 73.1

32.12 -51.3 49.25

-46.36 -87.38 -10.84

46.63 -90.89 -7.59

27.89 -83.43 50.26

-37.88 23.01 -1.74

// Vingerhoets, 2001

// Subjects=10

-39.19 -4.05 42.99

-13.07 -71.11 60.27

17.66 23.22 31.92

1.01 -15.05 57.51

54.77 -43.88 13.73

// Ng, 2001

// Subjects=12

26.06 -70.44 -5.9

-16.41 -58.5 55.27

-48.16 8.32 36.87

-48.37 24.19 22.98

57.23 13.77 -2.44

57.55 12.86 22.3

17.01 -62.2 48.34

-4.51 -61.65 55.38

-39.18 -38.37 53.67

36.18 -72.92 24.42

39.2 -74.57 6.61

57.53 16.06 21.98

-4.69 -56.41 42.54

20.2 -68.02 43.26

29.35 -82.13 -4.8

// Kucian, 2006

// Subjects=20

-46.83 -37.96 47.03

-40.94 -80.66 -8.23

40.69 -44.13 45.03

34.04 -85.9 23.5

23.79 -85.52 -17.82

-34.83 24.86 -16.54

43.45 11.34 18.2

55.37 21.47 22.6

43.31 32.19 11.66

36.49 31.8 -15.08

-44.01 8.15 23.37

24.2 -96.65 12.41

-34.71 -58.49 -23.98

// Zacks, 2002

// Subjects=24

-19.88 -60.62 -47.55

24.41 -56.1 -47.64

4.04 -35.37 -31.41

-31.5 -60.92 -27.16

-1.18 -78.89 -25.89

39.95 -62.82 -16.98

-59.52 -30.95 -15.08

-33.28 -86.5 -2.18

-.24 33.91 -13.54

-14.93 -72.3 -.54

44.56 -77.09 2.28

-13.96 -24.98 .36

15.23 -24.77 .97

10.04 -76.8 7.32

-47.5 -.32 1.86

24.87 8.55 .87

-23.74 9.54 2.72

-24.32 -78.66 23.78

34.02 -75.16 23.56

53.27 -21.81 19.08

-2.07 49.46 15.21

-56.88 -22.95 24.43

38 36.09 19.21

-49.03 -68.89 37.8

-62.03 -50.65 38.46

56.85 -61.93 37.55

-47.07 -.4 35.48

26.71 -64.5 46.16

3.89 -26.18 43.88

40.52 1.6 40.51

-45.77 -32.64 46.49

5.91 15.41 40.85

-28.38 -54.81 49.5

36.44 -37.04 52.25

-34.89 -10.18 56.41

0.82 1.3 62.51

// Carrillo, 2010

// Subjects=42

1.33 -36.86 10.24

-5.26 -45.36 -0.01

// O'Boyle, 2005

// Subjects=16

40.87 -53.73 57.18

-43.56 -45.39 47.71

24.39 -3.36 45.76

-21.04 9.24 45.28

59.76 14.35 26.59

21.49 -69.75 59.1

46.37 -64.38 -22.54

// Ecker, 2006

// Subjects=10

-37.1 -17.09 51.52

36.32 -5.13 49.09

-22.02 -5.34 50.1

6.01 10.71 48.04

27.94 21.55 -9.44

-18.36 -19.03 -4.63

56.47 11.8 22.42

-45.1 15.69 23.76

-37.1 -17.09 51.52

47.56 -62.48 -13.78

-41.99 -73.87 -4.41

-22.16 -71 25.23

32.91 -63.36 23.53

-22.04 -62.41 36.7

33.03 -54.77 35.01

// Prescott, 2010

// Subjects=8

30.89 3.36 48.34

-23.95 -8.04 67.21

56.5 7.65 23.95

-50.43 .89 26.44

11.24 21.5 36.8

34.39 -63.64 54.91

-30.44 -63.87 56.04

44.02 -51.15 50.15

-40.25 -52.51 51.72

49.51 -71.68 -31.95

-47.57 -77.47 -19.64

-20.7 -76.91 53.8

18.18 -86.65 50.74

33.99 -92.9 17.47

-20.95 -91.85 31.75

23.38 35.08 -25.26

-31.59 35.82 -14.32

59.52 42.13 13.76

// Ebisch, 2012

// Subjects=22

-44.11 31.44 19.95

-26.36 -63.49 36.88

-27.6 -76.75 21.41

-30.23 -56.16 -10.84

31.32 -46.37 -12.86

-50.08 -41.37 45.18

49.23 -41.64 36.8

4.67 25.21 30.94

// Kucian, 2005

// Subjects=22

-30.94 -84.08 12.1

44.08 -49.57 55.6

34.04 -85.9 23.5

40.69 -44.13 45.03

53.07 -76.93 -7.96

-55.56 -29.9 41.9

-33.85 -44.4 46.33

30.34 -83.92 -12.49

40.25 11.64 21.59

-40.75 4.97 23.63

20.54 -82.34 -18.08

43.32 38.78 13.25

-47.43 -77.5 -8.44

53.22 18.27 22.96

61.72 22.68 12.29

-5.24 -83.52 -6.32

-41.31 24.84 -16.43

43.07 -58.21 -25.33

46.8 -84.12 7.42

46.61 -83.34 -7.22

52.89 46.49 3.36

-24.24 -1.41 46.39

27.74 -80.21 38.74

33.33 29.12 -9.16

46.26 25.66 -12.4

-8.09 17.9 45.33

4.84 27.52 44.16

37.42 -45.52 41.86

44.04 -49.88 52.27

-46.85 -35.93 45.71

-52.41 -2.13 40.22

55.68 -23.23 38.22

-20.9 -67.41 40.54

24.08 -94.29 3.21

-40.1 -52.54 62.93

30.87 3.25 47.23

34.07 -82.4 26.52

27.79 -66.72 45.25

// Elizabeth, 2011

// Subjects=16

19.02 27.54 55.12

-13.43 33.71 53.94

15.3 61.1 23.85

-20.19 47.97 34.71

10.67 57.73 -1.4

1.7 -.69 52.62

-5.38 54.46 11.41

54.05 9.82 1.37

61.74 -2.3 9.16

-42.38 -21.7 59.91

2.6 -78.12 16.55

2.72 -36.69 34.86

58.76 -45.19 21.3

-53.94 -17.57 1.43

-52.45 -50.59 27.09

27.97 -48.28 -21.58

-37.96 -53.08 -23.34

24.59 -79.94 -38.56

-31.57 -80.04 -36.47
